# EV‐SPEC: Decoding the Spatial Proteomic Signature of the Extracellular Vesicle Corona

**DOI:** 10.1002/jex2.70151

**Published:** 2026-06-04

**Authors:** Camille Menaceur Vandenbroucke, Carsten Scavenius, Yuya Hayashi

**Affiliations:** ^1^ Department of Molecular Biology and Genetics Aarhus University Aarhus C Denmark; ^2^ Interdisciplinary Nanoscience Center (iNANO) Aarhus University Aarhus C Denmark

**Keywords:** endogenous protein corona, extracellular vesicle biogenesis, genetically engineered extracellular vesicles, proximity labelling, spatial proteomics

## Abstract

The concept of protein corona formation around extracellular vesicles (EVs) has given birth to new insights into how cells may recognize EVs by proteins presented at the EV surface. Here we present spatially resolved proteomics using the biotin ligase TurboID to map proteins interacting at the EV surface, without a need for physical isolation of the EV‐corona complexes. TurboID promiscuously biotinylates nearby proteins within a few nm distance from the fused ‘bait’ protein. We genetically engineered EVs by modifying CD63 as the bait protein to which TurboID was fused facing outward to map EV corona proteins. Biotinylated proteins were then analysed by Western blotting and liquid chromatography‐mass spectrometry. Western blots revealed protein patterns that are distinct depending on the localization of the fused TurboID. The mass spectrometry analysis identified many of the serum proteins commonly known to form a corona around a synthetic solid nanoparticle, also supporting those previously reported through the physical isolation approaches. Rather striking is, however, the EV corona footprint of endogenous proteins that tells us about the EV biogenesis and what the intrinsic endogenous corona might look like. This approach, which we coined EV‐SPEC (Spatial Proteomics of Endogenous Corona), has thus the potential to revolutionize our understanding of EV biology by shifting the focus from the EVs themselves to the proteins that make up the corona, or how the cell ‘sees’ them, in analogy with the biomolecular corona extensively characterized for synthetic nanoparticles.

## Graphical abstract

This study introduces ‘EV‑SPEC’, a spatial proteomics method using TurboID fused to the transmembrane protein CD63 to map the protein corona of extracellular vesicles (EVs) without physical isolation. We show that EV‐SPEC identifies the protein‐protein interaction footprint of serum proteins, similar to those typically found on synthetic nanoparticles, as well as endogenous proteins linked to EV biogenesis. Our findings provide new insights into how EV‐protein corona may evolve from biogenesis to external acquisition of serum proteins in the extracellular space.

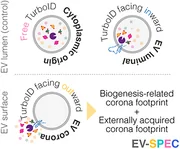

## 1 Introduction

It was in 2010 when a sensational article entitled ‘*What the cell ‘sees’ in bionanoscience*’ beautifully conceptualized complex biophysical interactions between biomolecules and nanoparticles, with the bold implication for their pattern recognition by surface receptors (Walczyk et al. 2010). Proteins are among those that form a ‘corona’ around the nanoparticle exhibiting a biologically meaningful residence time at the surface long enough for triggering, for example, receptor‐mediated endocytosis (Walczyk et al. 2010). Since then, most (if not all) of the studies in the relevant field have adopted such a concept, and 10+ years of evidence supports that cells indeed recognize the proteins adsorbed to nanoparticles rather than the bare surface (Ren et al., 2022, Mohammad‐Beigi et al. 2020b). Emerging in the rapidly growing field of extracellular vesicles (EVs) is a question of whether this holds true for EVs, or biological nanoparticles in contrast to the synthetic ‘hard‐surface’ counterpart.

EVs are continuously exposed to a variety of proteomic repertoires that biological systems exhibit, for example, during EV biogenesis, following release into the extracellular space, and after uptake by recipient cells (Buzas 2023). Since the nanoparticle‐protein corona may retain the footprint of its inter/intracellular journey (Tóth et al. 2021, Hayashi et al. 2013, Bertoli et al. 2016, Hayashi et al. 2017, Wang et al. 2021), profiling of EV‐protein corona at a given time is likely very challenging both technically and conceptually. Recent efforts have nonetheless indicated that the composition of this corona is dynamic and context‐dependent, presumably playing a decisive role in the biological fate of EVs by modulating their stability, biodistribution, interaction with, and uptake by target cells (Mohammad‐Beigi et al. 2020b, Mohammad‐Beigi et al. 2020a, Wolf et al. 2022, Dietz et al. 2023). Far from being an artefact, this protein interface may represent a key functional regulator of EV biology.

There is, however, a huge obstacle that has delayed the adoption of the protein corona theory from synthetic nanoparticles; that is, EVs themselves are made of biomolecular complexes. It is therefore hard to imagine proteins being ‘non‐specifically’ strongly bound (by denaturation) at the membrane surface of EVs, but rather likely undergoing soft protein‐protein interactions to form a corona. Soft interactions are very difficult to study as the proteins are easily washed away, whereas hard interactions such as those occurring at solid nanoparticles can be pulled down simply by centrifugation of the nanoparticles. For instance, conventional isolation and analytical approaches adapted to EVs are often too harsh or insufficiently specific, disrupting the native corona structure and complicating the distinction between intrinsic EV proteins and transiently adsorbed contaminants (Liam‐Or et al. 2024, Buzas 2023). Furthermore, the specificity of corona protein identification is often challenged in physical isolation approaches when serum is used without depletion of serum‐derived EVs (Gardiner et al. 2016, Urzì and Crescitelli 2022). Lessons learnt from the nanoparticle‐protein corona field therefore tell us that physical isolation of the complexes is never enough to gain insights into the biological identity conferred by the corona as a whole including those considered ‘soft’ and ‘short‐lived’.

One of the breakthroughs for synthetic nanoparticles is a click chemistry approach to arrest soft interactions of proteins at nanoparticles via crosslinking to strongly bound proteins (Mohammad‐Beigi et al. 2020a). Likewise, proximity biotinylation has become a popular labelling method for proteins interacting with a bait protein fused with biotin ligase such as TurboID, a fast‐reacting mutant derived from BioID facilitating their enrichment and subsequent identification by mass spectrometry (Tassoni et al. 2025, Branon et al. 2018, Roux et al. 2018). Coincidentally, its effective range is similar to click chemistry used for studying the protein corona: a few nanometres from the bait protein. Indeed, the proximity labelling matches very well with the fact that EVs are biological nanoparticles. If TurboID is fused to an EV surface protein, proximity labelling of proteins around the bait occurs as the EVs are formed without a clear boundary of protein‐protein interactions before and after the bait protein's localization to the EV membrane. We, however, consider proteins that potentially interact with the bait at the molecular level would do so at any point of the EV's lifetime. The footprint of protein‐protein interactions revealed by such an approach should thus provide a catalogue of proteins that potentially contribute to the biological identity of EVs, notably, adding another dimension onto those uncovered by physical isolation methods.

The aim of this work is therefore to develop a TurboID‐based protocol for decoding the spatial signature of the EV‐protein corona in isolation from the EV's protein cargo on the luminal side. This approach offers unprecedented spatial resolution to investigate the molecular microenvironment of EVs with implications for their origin, recognition, uptake, and biological functions. Here we report our proximity labelling method to map potential EV corona proteins, both endogenous and externally acquired, with a particular emphasis on methodological considerations that arose during the method development phase. Using the optimized protocol, we find that some of the proteins identified are commonly known to form a corona around synthetic nanoparticles, further supporting those previously reported through conventional physical isolation approaches. Importantly, our analysis distinguishes between corona protein footprint of endogenous origin, characterized by the presence of human‐derived proteins associated with EV biogenesis, and an acquired corona formed in the extracellular milieu, as evidenced by the identification of bovine serum proteins among the detected hits. To highlight the intrinsic and endogenous component of the EV‐protein corona revealed, we term this method EV‐SPEC (Spatial Proteomics of Endogenous Corona) with an implication in its future use in an in vivo setting. Our findings underpin the dynamic and composite nature model of the EV corona, shaped both by intrinsic vesicle‐associated components and by extrinsic protein adsorption from the surrounding environment.

## 2 Materials and Methods

### 2.1 Plasmid Construction

All plasmids were constructed using GeneArt Gibson Assembly Kits (Invitrogen) by assembling DNA fragments amplified from plasmids containing TurboID or CD63; V5‐TurboID‐NES_pCDNA3 was a gift from Alice Ting (Addgene plasmid #107169) and pCMV‐Sport6‐CD63‐pHluorin was a gift from DM Pegtel (Addgene plasmid #130901). All constructs used in this study are listed in Table S1 with sequence details and also available from the authors upon request.

### 2.2 Mammalian Cell Culture and Transfection

HEK293T cells (passage under 15) were maintained in Dulbecco's Modified Eagle Medium (DMEM) supplemented with GlutaMAX (Gibco), 10% (v/v) fetal bovine serum (FBS) and 1% (v/v) anti–anti (Gibco), under standard culture conditions (37°C, 5% CO_2_, humidified atmosphere). Cells were passaged at ∼70%–90% confluency. All cultures were routinely monitored for morphology and confluency, and confirmed to be free of mycoplasma contamination. HEK293T cells were transfected using Lipofectamine 3000 (Invitrogen) following the manufacturer's instructions. The DNA–lipid complexes were incubated for 10–15 min at room temperature (RT, 21°C) before being added dropwise to each well containing cells at approximately 60% confluency in fresh complete DMEM. Cells were returned to the incubator (37°C, 5% CO_2_) and maintained for 48 h before downstream analysis.

### 2.3 Proximity Labelling by TurboID (Optimized Protocol)

For proximity biotinylation, cells incubated in the complete cell culture medium under transfection were further supplemented with 50 µM ATP and with or without 50 µM biotin for the last 16 h. The conditioned medium was centrifuged at 300 rcf for 3 min at RT to remove cell debris. The supernatant was retained for subsequent immunoprecipitation (IP), while the cell pellet was lysed in ice‐cold RIPA buffer supplemented with cOmplete Mini Protease Inhibitor Cocktails (Roche). The supernatant was centrifuged at 2000 rcf for 10 min at 4°C, and the resulting pellet was discarded. The supernatant was then centrifuged at 5000 rcf for 10 min at 4°C to remove residual debris. For samples supplemented with biotin, the final supernatant fraction was transferred onto a Zeba Dye and Biotin Removal Spin Column (Thermo Scientific) to remove free biotin. Triton X‐100 (final 1%) was added to the sample to lyse EVs. Prior to IP, the total protein concentration of each condition was quantified using a Pierce Rapid Gold BCA Protein Assay Kit (Thermo Scientific). For each sample, the Pierce Streptavidin Magnetic Beads (Thermo Scientific) slurry was thoroughly resuspended by pipetting, and the beads were diluted and washed twice in wash buffer (TBS‐Tween with a cOmplete protease inhibitor cocktail). The sample was incubated with the beads for 1 h at 4°C on an end‐over‐end rotator. After incubation, the flow‐through from captured beads was carefully collected and stored at −70°C until analysis (FT samples). Beads bound by biotinylated proteins were then washed three times in wash buffer. At the final wash, the bead suspension was transferred to a new tube to minimize carryover of non‐specific proteins adsorbed to the tube wall. After magnetic separation, the wash buffer was removed, and the beads were directly resuspended in 1x Bolt LDS Sample Buffer pre‐mixed with Bolt Sample Reducing Agent (Invitrogen). Samples were vortexed and incubated for 10 min at 70°C. Beads were then recaptured, and the flow‐through was transferred to a Protein LoBind tube (Eppendorf) and stored at −70°C (IP samples). All other protein samples (cell lysates and FT samples) were mixed with reducing LDS sample buffer likewise as per the supplier's instruction.

### 2.4 Western Blots

Proteins were separated on a Bolt 4–12% Bis‐Tris plus Mini Protein Gel (Invitrogen) with Bolt MES SDS Running Buffer (Invitrogen) following the manufacturer's instructions. Loading volumes were adjusted based on BCA assay results to normalize protein mass. Following SDS‐PAGE, proteins were transferred onto nitrocellulose membranes using Bolt Transfer Buffer (Invitrogen) according to standard procedures. Membranes (8.5×8.5 cm) were briefly rinsed in water, soaked in the transfer buffer, and assembled with pre‐soaked blotting pads and Whatman papers. Transfer was performed at 30 V for 1 h. Membranes were then rinsed with deionized water, briefly stained with phenol red for 10 min to verify transfer efficiency, and washed three times with water. Blocking was carried out for 1 h at RT in blocking buffer (TBS‐Tween containing 5% (w/v) BSA). For streptavidin Western blots, membranes were incubated for 1 h with Pierce High Sensitivity Streptavidin‐HRP (Thermo Scientific) diluted 1:7000 in blocking buffer. For all other protein targets, membranes were incubated with the primary antibodies either for overnight at 4°C (see Table S1 for antibody references and dilutions). Following three washes of 5 min each, membranes were incubated with the appropriate secondary antibodies for 1 h at RT. After three additional washes of 5 min each, chemiluminescence was developed using a Pierce ECL plus Western Blotting Substrate (Thermo Scientific), and signals were captured with an ImageQuant LAS4000 system (GE Healthcare).

### 2.5 EV Isolation, Characterization and Ex Situ Proximity Labelling

Forty‐eight hours after transfection, EVs were isolated from cell culture supernatants. Supernatants were first cleared by differential centrifugation at 4°C to remove cells and debris: 500 rcf for 3 min, 2000 rcf for 10 min, and 5000 rcf for 10 min. EVs were then diluted in PBS and pelleted by ultracentrifugation at 100,000 rcf (maximum) for 2 h at 4°C. The resulting EV pellet was gently resuspended in 100 µL of 0.22 µm‐filtered 1% (w/v) BSA in PBS for nanoparticle tracking analysis (NTA) and Western blotting (as described above), or in 1 mL of complete cell culture medium supplemented with 50 µM ATP and with or without 50 µM biotin for ex situ proximity labelling. In NTA, EV size and concentration were analysed using a ZetaView Evolution system (Particle Metrix GmbH, Germany) and its software (Version 1.0.4.6). Samples, diluted in 0.22 µm‐filtered PBS to 10^7^–10^9^ particles/mL, were introduced into the analysis chamber after calibration with 100 nm standard beads. For each condition, two 30 s runs were recorded at 11 positions at RT. Particles were tracked by the Brownian motion, and the hydrodynamic diameter was calculated using the Stokes–Einstein equation. For ex situ proximity labelling, resuspended EVs were incubated for 1 h at 37°C. Free biotin was subsequently removed using a Zeba Dye and Biotin Removal Spin Column (Thermo Scientific), and the EVs were lysed in 1% Triton X‐100 for IP to capture biotinylated proteins as described above.

### 2.6 Sample Preparation and Mass Spectrometry Analysis

After the third wash of IP, instead of reducing LDS sample buffer, S‐Trap lysis buffer (5% SDS in 50 mM Tris‐HCl, pH 8.5) was directly added to the beads and the SDS removed using a S‐Trap Micro Spin Column (ProtiFi, NY, USA) according to the manufacture's recommendation (HaileMariam et al. 2018). Samples were digested using sequence grade trypsin (Proteomics grade, Sigma‐Aldrich). The tryptic peptides were lyophilized and dissolved in 0.2% formic acid. LC‐MS/MS was performed in on an EASY‐nLC 1200 system (Thermo Scientific) connected to an Orbitrap Eclipse Tribrid Mass Spectrometer (Thermo Scientific) with a 2 cm trap column (100 µm i.d.) and a 20 cm analytical column (75 µm i.d.), both packed in‐house with ReproSil‐Pur C18‐AQ 3 µm resin (Dr. Maisch GmbH, Germany). Peptides were eluted at 250 nL/min using an 115 min gradient from 5%–44% phase B (0.1% formic acid and 80% acetonitrile), followed by 100% phase B for 5 min. Protein identification and quantification were performed using Proteome Discoverer 2.5 (Thermo Scientific). Data were searched against the human and bovine reference proteomes (uniprot.org) using the Sequest search engine with the following parameters: MS error tolerance of 10 ppm, MS/MS error tolerance of 0.02 Da, trypsin as the protease with two missed cleavages, and carbamidomethylation as a fixed modification. Label‐free quantification was based on precursor ions. The mass spectrometry proteomics data have been deposited to the ProteomeXchange Consortium via the PRIDE partner repository with the dataset identifier PXD077319 (Perez‐Riverol et al. 2022). Raw peptide abundance values were used for clustering analysis with a heatmap after log2‐transformation and standardization.

### 2.7 Bioinformatic Analysis

For heatmap visualization and unsupervised hierarchical clustering of the scaled dataset, the distance matrix was calculated using Ward's minimum variance algorithm with the Euclidean metric using the packages gplots (ver. 3.1.3.1) and dendextend (ver. 1.17.1) in the R environment (ver. 4.4.0). Heatmaps are shown with or without a threshold on peptide abundance, but for the downstream bioinformatic analysis, only proteins with a minimum peptide abundance of 2,000,000 in at least one of the samples (approximately half of the total protein hits) were included in order to exclude low abundance proteins. Welch's one‐way ANOVA (analysis of variance) was performed in R on log2‐transformed and/or standardized data, followed by Games‐Howell post hoc pairwise comparisons with a significance level of *α* = 0.05. For gene ontology analysis, the list of identified proteins was analysed using the STRING database (Search Tool for the Retrieval of Interacting Genes/Proteins) to explore functional associations and enriched biological processes, with a minimum confidence threshold of 0.7 for the combined score. Enrichment was assessed for biological process, molecular function, and cellular component categories, using the full *Homo sapiens* and *Bos Taurus* proteomes as the reference background.

## 3 Results

### 3.1 Optimization of the Experimental Workflow—Biotin

The initial protocol builds on several approaches already described in the literature, but its originality lies in the expression of TurboID directly at the surface of EVs via fusion to a transmembrane protein (Li et al. 2023). This strategy leverages proximity biotinylation to selectively label proteins that are in close proximity to the EV surface, thereby enabling robust identification of the components of the protein corona. To this end, HEK293T cells were transfected to express different CD63‐TurboID fusion variants. The protocol was developed using the CD63‐TurboID (EC1) construct, in which TurboID is inserted into the first extracellular loop of CD63, positioning the enzyme on the external surface of the vesicles (Figure 1A). This design builds on the work of Verweij and colleagues, who demonstrated that fusion of pHluorin, a pH‐sensitive EGFP variant, to the EC1 loop of CD63 enables vesicle tracking while largely preserving proper trafficking and incorporation into secreted EVs (Verweij et al. 2018). Using advanced imaging approaches, they further confirmed the correct localization of tagged CD63 within EV membranes and showed that fusing reporters within the EC1 loop does not perturb vesicle morphology or targeting. Their findings indicate that this region of CD63 tolerates insertions without affecting EV biogenesis or function, making it a suitable site for reporter engineering (Verweij et al. 2018, Verweij et al., 2021). Following this rationale, we envisioned positioning TurboID within the EC1 loop enables the enzyme to biotinylate proteins that interact with, or reside in the immediate microenvironment of the outer EV surface, thereby extending proximity‐labelling approaches to the extracellular interface of EVs. Although CD63 is classically described as a late endosome marker, multiple studies have demonstrated its strong enrichment in EVs, often exceeding its levels at the plasma membrane. Quantitative proteomic analyses and imaging studies have confirmed that CD63 is consistently associated with EV membranes across diverse populations, making it a robust EV marker (Kowal et al. 2016, Jeppesen et al. 2019). In line with MISEV 2018/2023 guidelines, CD63 is considered a transmembrane or lipid‐bound protein commonly used for EV characterization (Théry et al. 2018, Welsh et al. 2024). Its widespread presence in EVs, combined with its tolerance for protein fusions, makes CD63 an ideal scaffold for anchoring TurboID and enabling efficient proximity labelling at the EV surface.

**Figure 1:**
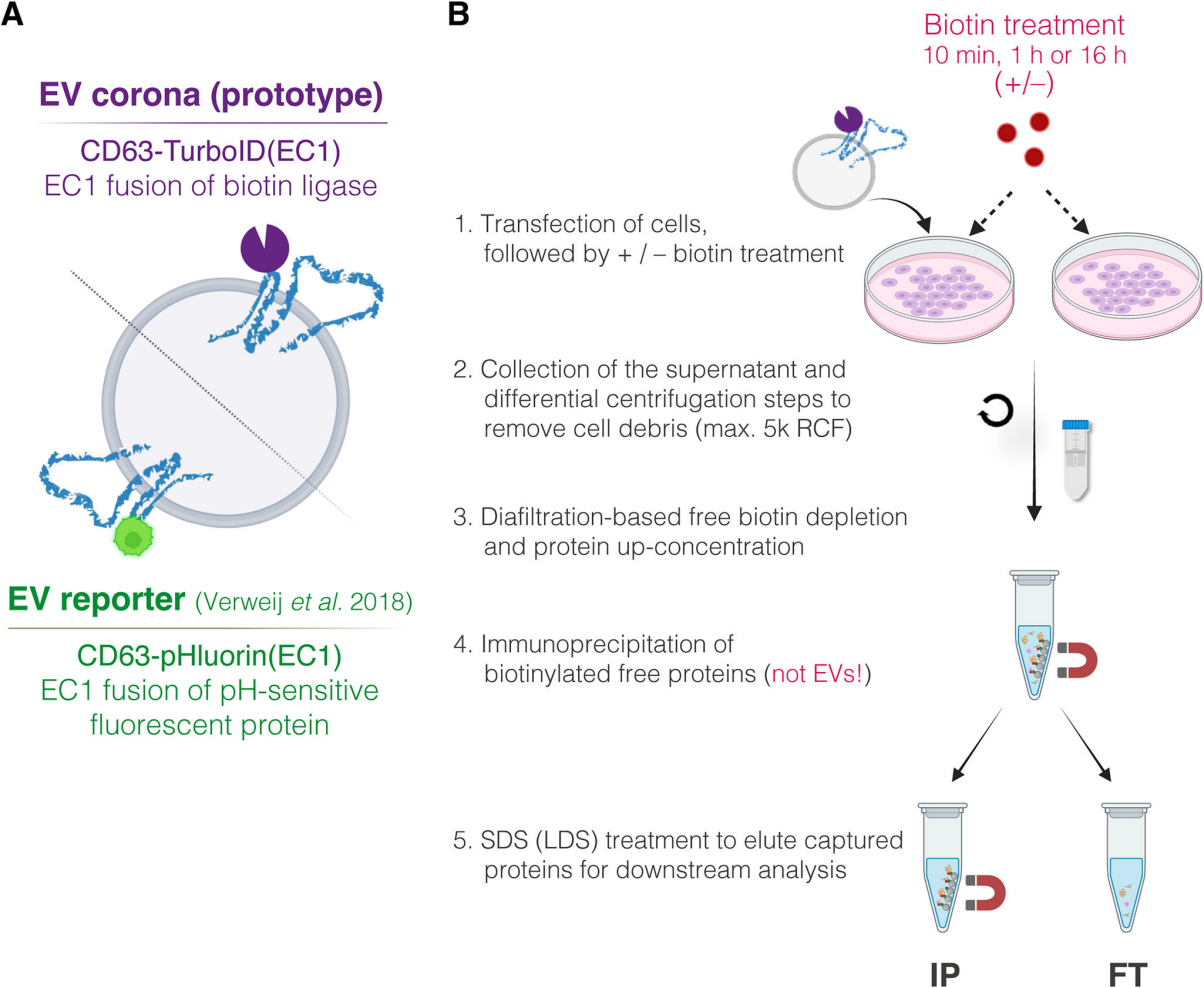
Strategy for labelling the surface of EVs using TurboID. (A) Following the CD63‐pHluorin topology (inspiration from Verweij and colleagues), TurboID is inserted into the first extracellular loop of CD63 at the EV membrane. This orientation places the ligase in direct contact with extracellular proteins, enabling their proximity‐dependent biotinylation. (B) Schematic diagram of the experimental protocol: HEK293T cells are transfected to express CD63‐TurboID(EC1), then incubated in the presence of biotin for different periods of time (10 min, 1 h or 16 h) before processing of the supernatants by differential centrifugation, free biotin depletion, and recovery of biotinylated free proteins (not EVs) by immunoprecipitation on streptavidin beads. The proteins bound to the beads (IP) are then eluted and analysed by Western blotting, while the flow‐through (FT) contains the proteins that were not retained. Figure created with BioRender.com.

The experimental workflow (Figure 1B) was organized as follows. Cells were cultured under standard conditions prior to transfection with a newly designed plasmid construct for overexpression of CD63‐TurboID (EC1). After 24 h of expression, biotin was added to the cell culture medium to enhance the activity of TurboID, inducing biotinylation of proteins in the immediate vicinity of CD63. Multiple labelling durations were initially tested (10 min, 1 h, and 16 h), and a non‐transfected wild‐type (WT) control was included to assess nonspecific background biotinylation. Following incubation with biotin, supernatants were collected and cleared through differential centrifugation steps to remove cells and debris. Biotinylated proteins in the resulting supernatant were then concentrated while depleting free biotin by 3 kDa cut‐off diafiltration, and subsequently enriched by immunoprecipitation using streptavidin‐conjugated magnetic beads. For analysis, samples were divided into two fractions: (i) The immunoprecipitated fraction (IP), containing biotinylated proteins captured by the beads, and (ii) the flow‐through fraction (FT), containing proteins that were not captured. It is important to note that this study focuses on free proteins carrying the biotin signature of proximity interaction with EVs, rather than on physical isolation of intact EV–protein complexes. Accordingly, purification conditions can be adjusted to efficiently remove nonspecific contaminants without affecting the proteins of interest. By contrast, studies focusing on native EV‐protein complexes must avoid harsh washing steps, which can disrupt the corona and lead to the loss of weakly bound components. As highlighted by Tassoni and colleagues, preserving corona integrity requires carefully optimized protocols, including gentle washes with buffers such as TBS‐T, milder elution conditions, and strict standardization of experimental parameters to ensure reproducible, interpretable, and reliable results (Tassoni et al. 2025). Finally, both fractions were analyzed by Western blotting to compare the protein profiles obtained (Figure 2). In the first set of experiments, to our surprise, no proteins were detected in IP samples with a biotin treatment but only in those without biotin addition (Figure 2A, left). In contrast, FT samples revealed a pattern of proteins in particular in the longest incubation time Figure 2A, right). This highlights a unique challenge associated with TurboID‐mediated labelling in the extracellular space. Unlike intracellular labelling, where unincorporated biotin can be effectively removed by washing cells prior to lysis (Branon et al. 2018, Cho et al.), excess free biotin remains in the cell culture medium, eventually competing with biotinylated proteins for binding to streptavidin beads despite the diafiltration step to deplete free biotin. This competition causes premature bead saturation and dramatically reduces IP efficiency. Addressing this issue may require optimized strategies to either completely remove or neutralize free biotin in the supernatant prior to IP, such as dialysis or buffer exchange, which are less critical in the original intracellular TurboID applications.

**Figure 2:**
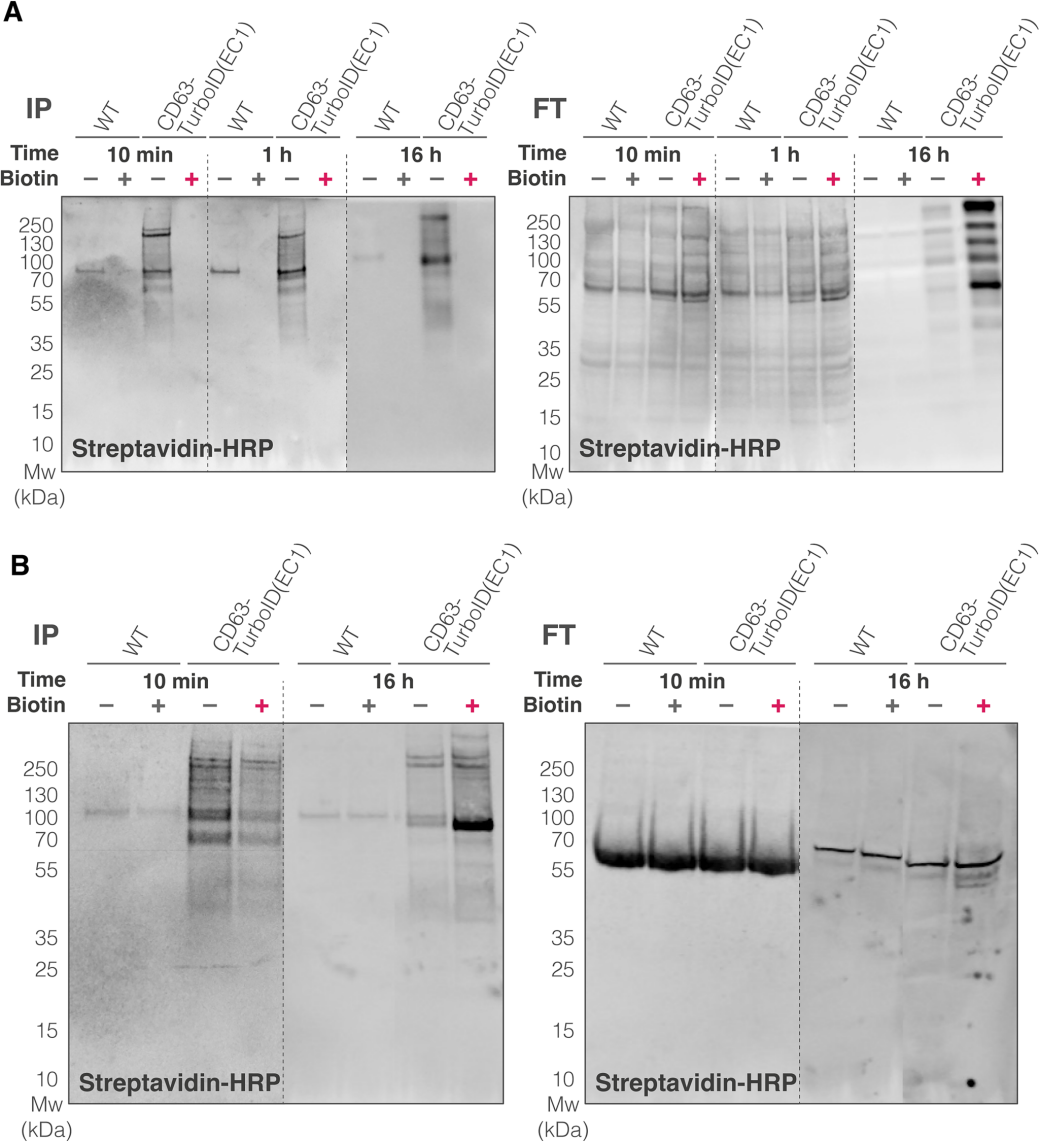
Optimization of the TurboID‐based proximity labelling protocol—biotin: Western blot analysis on WT and CD63‐TurboID (EC1) conditions. The biotin+ condition on the transfected samples are marked in magenta. (A) Results obtained on the IP and FT fractions from the initial protocol presented in Figure 1. (B) Results obtained for the IP and FT fractions after replacing a free biotin removal step from 3 kDa cut‐off diafiltration to biotin removal spin columns.

To overcome this saturation issue, we used biotin removal columns to bind the excess free biotin present in the samples. Eliminating the unincorporated biotin frees the binding sites of streptavidin and thereby improves the capture of biotinylated proteins. This dedicated purification step, replacing diafiltration, enhanced the specificity of the labelling and drastically increased the IP efficiency, ensuring that the recovered proteins truly correspond to those biotinylated by TurboID (Figure 2B). Moreover, consistent with the earlier observation, the band intensities were superior after the longer incubation time (16 h). This incubation time was therefore selected for the remainder of the study.

### 3.2 Optimization of the Experimental Workflow—ATP

To further optimize our proximity biotinylation protocol, we considered the enzymatic constraints inherent to TurboID. TurboID is a biotin ligase whose catalytic activity requires ATP hydrolysis (Cho et al.). In our experimental configuration, TurboID is fused to EVs and located in the extracellular compartment. However, ATP concentrations outside the cytoplasm are extremely low, which could limit the efficiency of proximity biotinylation. To address this limitation and support enzyme activity, we supplemented the cell culture medium with ATP at the same time of biotin addition to better mimic the intracellular environment in which TurboID normally operates. Indeed, TurboID is adapted to the cytoplasmic ATP concentration, which typically lies in the millimolar range (approximately 1–5 mM). However, the results were negative with no visible bands: although within the physiological cytoplasmic range, the ATP concentration we initially used (1 mM) may be cytotoxic when applied extracellularly, as reported in other cell types (for instance, 1 mM induces approximately 20% cell death in bovine monocytes) (Woo et al.^2007^, Feng et al.^2020^). Based on this, we repeated the experiment using 1 or 50 µM ATP, a more moderate and physiologically compatible concentration. At this point, we also noticed a consistent slight decrease of the protein concentration in the biotin+ samples which go through the biotin removal step. To enable proper comparison across conditions, total proteins were quantified prior to IP, and the analyses were carried out using equivalent protein loads. Addition of 50 µM ATP produced a clear and distinct protein profile, demonstrating efficient biotin labelling under these conditions and validating the experimental protocol (Figure 3).

**Figure 3:**
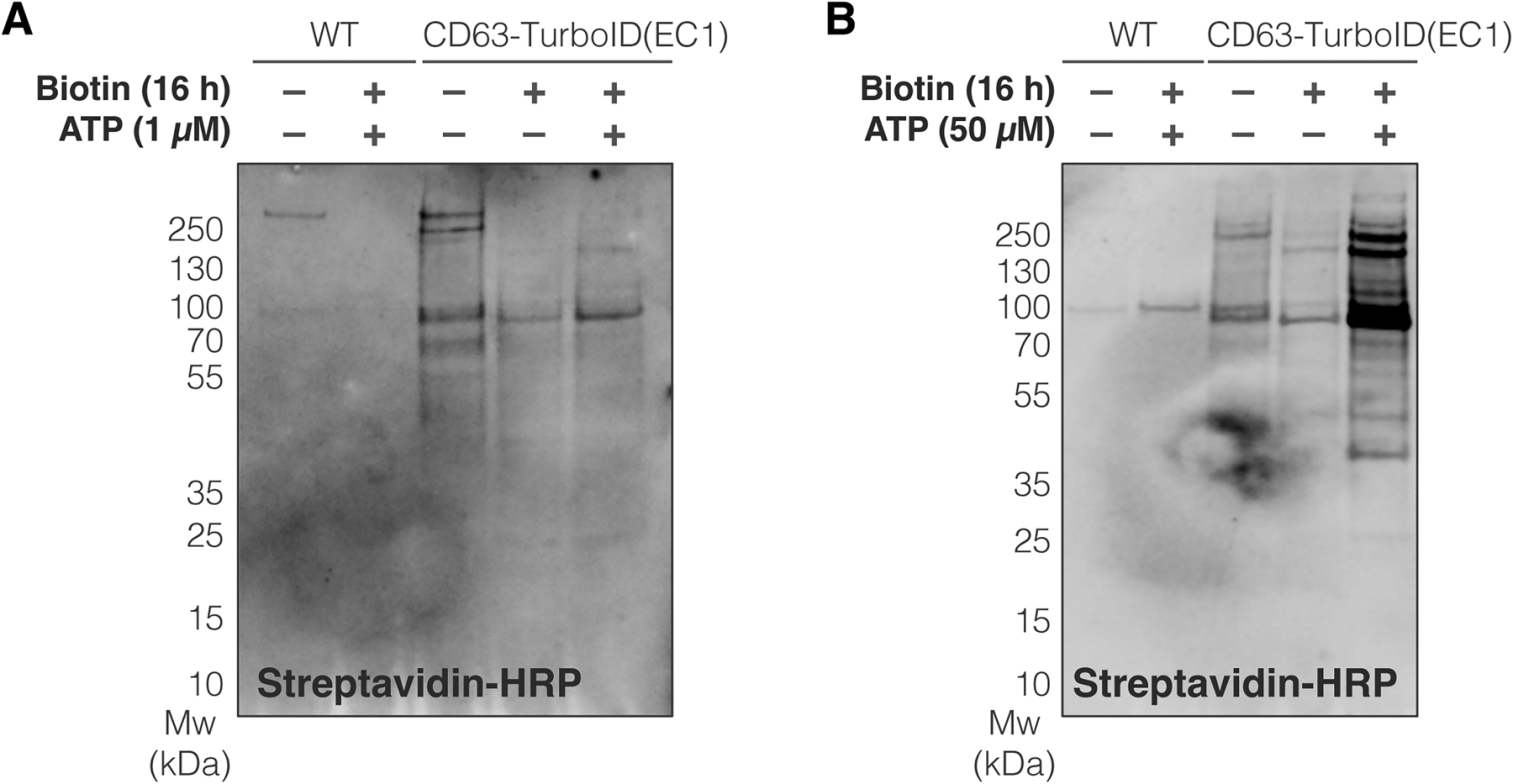
Protocol optimization with ATP treatment. The cells were treated with 1 µM (A) or 50 µM (B) of ATP during the biotin treatment (16 h) and analysed by Western blot.

The final, optimized protocol integrates the two revised steps described above: the use of biotin removal spin columns to efficiently deplete excess biotin and moderate ATP supplementation to enhance TurboID activity (Figure 4). We also added a detergent treatment step (1% Triton X‐100) prior to IP in order to ensure capture of biotinylated proteins carried inside EVs to be discussed below. Using this refined workflow, we were able to comprehensively characterize the protein profiles associated with the different CD63‐TurboID constructs.

**Figure 4:**
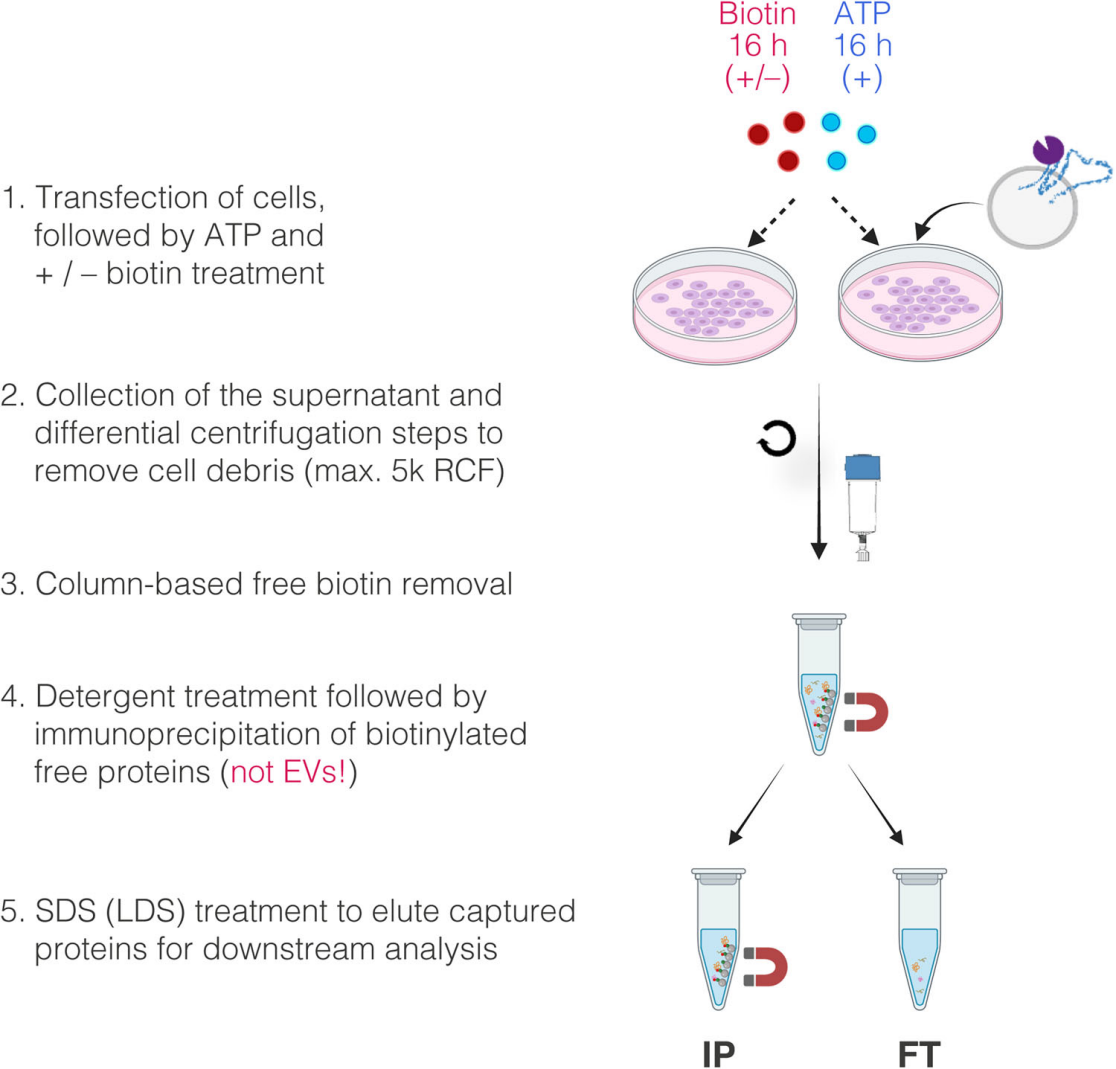
Schematic diagram of the optimized protocol for proximity biotinylation based on CD63‐TurboID (EC1). After transfection, the cells are incubated for 16 h with biotin (50 µM) and ATP (50 µM), the supernatants are cleared by differential centrifugation, and then free biotin is removed by spin columns. The eluates (mixture of biotinylated proteins and EVs) are first lysed with 1% Triton X‐100 and then captured by immunoprecipitation using streptavidin beads, washed and eluted from the beads for analysis by Western blotting. Figure created with BioRender.com.

### 3.3 Exploration of the EV‐Protein Corona Using Various CD63‐TurboID Constructs

In the subsequent experiments, we used multiple CD63‐TurboID fusion variants as shown in Figure 5.

**Figure 5:**
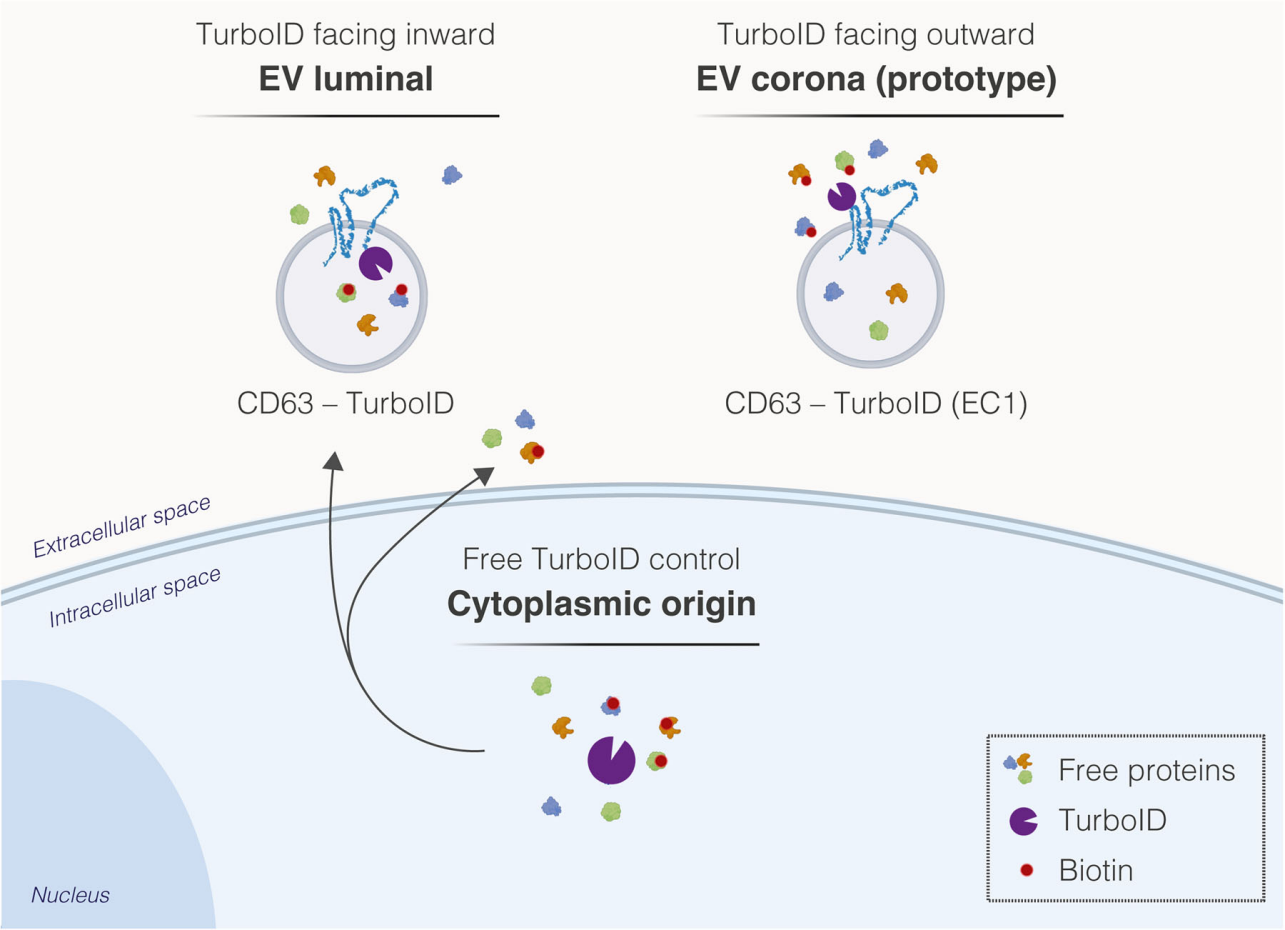
Conceptual diagram of CD63‐TurboID constructs used in this study. The CD63‐TurboID construct positions TurboID on the luminal side of the EV membrane ('EV luminal'), enabling partial proximity labelling of intraluminal proteins. The CD63‐TurboID (EC1) variant carries TurboID fused to the extracellular domain of CD63, oriented towards the outside of the vesicle to label surface proteins ('EV corona'), while TurboID alone expressed in the cell serves as a cytosolic biotinylation control for biotinylated proteins directly secreted and/or actively/passively loaded into EVs ('Cytoplasmic origin'). Note that the EV corona variant shown here is a prototype and replaced by yet another construct discussed below. Figure created with BioRender.com.

The CD63‐TurboID (EC1) variant was initially described and used for the development of the experimental protocol. In the CD63‐TurboID variant, TurboID is fused to the C‐terminal end of CD63 and therefore localized inside the EV. A previous study showed that CD63's EV sorting efficiency strongly depends on the tag's position (Corso et al. 2019). The authors observed that C‐terminal fusions of CD63 better preserve the protein's ability to incorporate into EVs, resulting in more homogeneous loading and several copies per vesicle comparable to or higher than N‐terminal fusions. Conversely, N‐terminal fusions could disrupt key interactions involved in endosomal trafficking and vesicle biogenesis, leading to less efficient or more variable incorporation (Sulaiman et al. 2025). Based on those quantitative results at the molecule‐per vesicle level, positioning the protein of interest at the C‐terminal of CD63 is more appropriate to maximize its targeting to EVs while preserving CD63's structural and sorting functions (Corso et al. 2019). This variant serves as a control for labelling internal cargo proteins only, as biotinylation is limited to the vesicular lumen without access to free proteins on the extracellular side. As a control condition for experimental background we used TurboID without the bait protein. TurboID expressed without vesicular anchoring remains soluble in the cytoplasmic compartment and does not associate with EVs. However, it does not prevent the release of already biotinylated proteins from the cytoplasm as a part of the physiological protein secretion process—be it as a secretory protein or following active/passive loading into EVs. The labelling pattern should therefore be stochastic in nature due to the absence of the bait protein, but the detection of biotinylated proteins in the cell culture supernatant may reflect a pattern of physiological protein/EV secretion. In Western blots, three distinct protein profiles were obtained from the different TurboID conformations (Figure 6A). However, the profile associated with the CD63‐TurboID (EC1) construct proved particularly difficult to obtain, repeatedly leading to lower signals than the other two control variants (note that the earlier Western blots required longer colour development times to visualize proteins biotinylated by the prototype EV corona construct). After repeating the experiments and consistently observing the same phenomenon, we concluded that this configuration was likely suboptimal. It is plausible that the insertion of TurboID at the EC1 loop of CD63 disrupts protein folding at the EV membrane due to excessive steric hindrance. This hypothesis is supported by the biophysical properties of the protein: TurboID is a relatively large enzyme of approximately 35 kDa, nearly 30% bigger than superecliptic pHluorin (the model protein used to design CD63‐TurboID). Moreover, TurboID (of the BirA family) adopts a conformation in which the N‐ and C‐termini are positioned far apart (Figure 6B). In contrast, EGFP, the structural ancestor of superecliptic pHluorin, forms a compact β‐barrel in which both termini emerge in proximity (Figure 6B). This marked difference in size and three‐dimensional architecture strengthens the idea that TurboID, when inserted into the EC1 loop, may introduce substantial steric constraints, thereby impairing the enzyme activity or surface presentation of CD63 on EVs.

**Figure 6:**
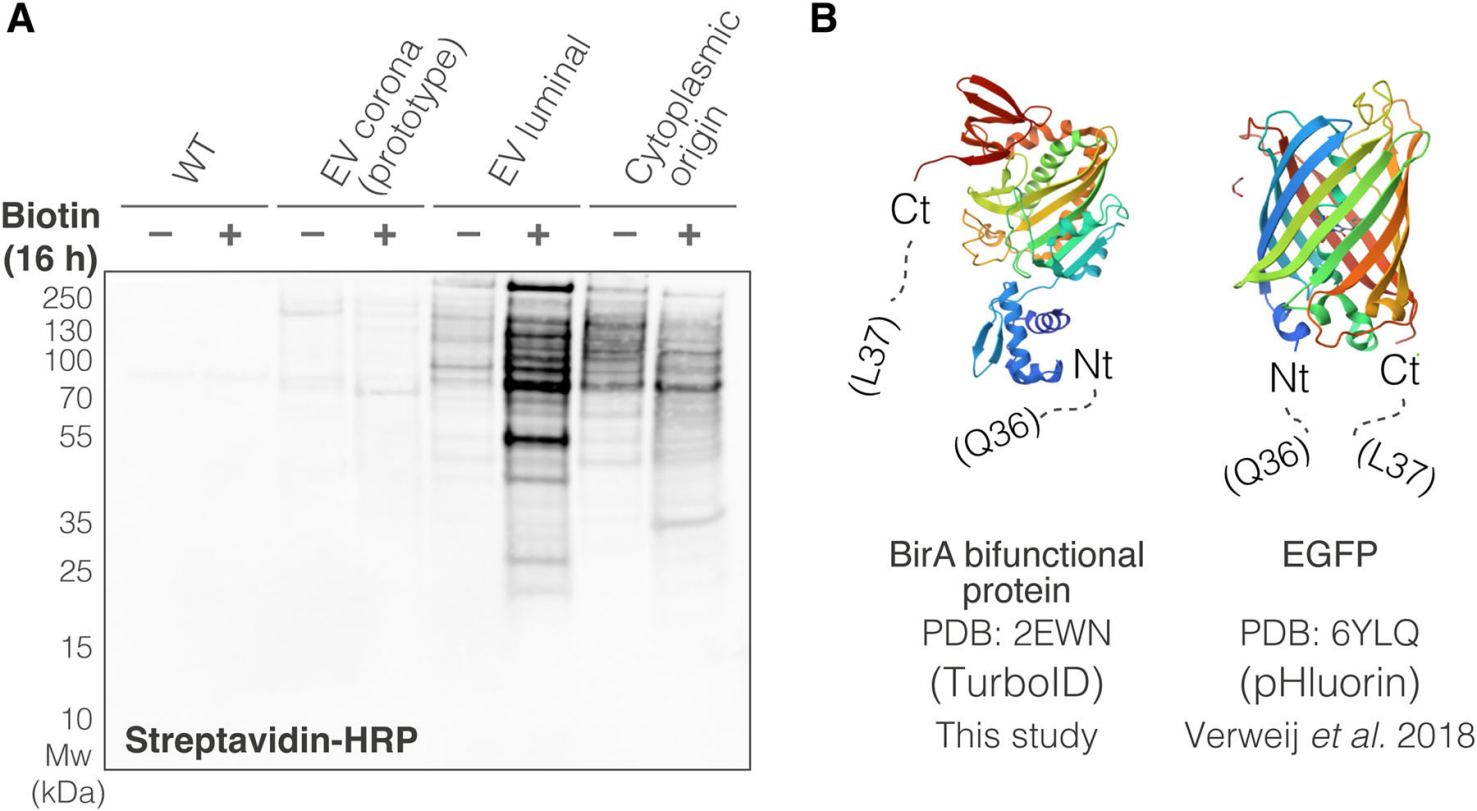
Validation of the three CD63‐TurboID fusion variants. (A) Western blot results on the three constructs using the optimized protocol. (B) Three‐dimensional crystal structures of fu proteins. Structural analysis reveals that the N‐ and C‐termini of BirA are distant from each other than those of EGFP. The amino acid residues with the dashed line extensions indicate the fused position in CD63.

### 3.4 Truncated CD63 as an EC2‐Less Scaffold for Effective Outward TurboID Presentation

Based on the work of Curley and coworkers, we adopted a modified configuration involving truncation of the C‐terminal domain at the second extracellular loop (EC2) that still preserves its localization to the EV membrane (Curley et al. 2020), and termed its fusion to TurboID as CD63 (Δ112–238)‐TurboID. Recent studies suggest that truncation of the C‐terminus, rather than the N‐terminus, enhances construct stability and reduces the risk of mislocalization or cleavage of the fused protein, thereby ensuring proper and stable integration into the EV membrane (Sulaiman et al. 2025, Curley et al. 2020, Nørdgård et al. 2022). Crystallographic data of tetraspanins, including the work of Zimmerman and colleagues on CD81, show that EC2 is the most variable region both in sequence and in conformation (Zimmerman et al. 2016). This variability is accompanied by substantial structural flexibility, suggesting that the functional integrity of tetraspanins relies only marginally on the distal portion of EC2, which also harbours a different degree of glycosylation sites. In this context, truncation of CD63 immediately after the transmembrane domain 3 (TM3) is therefore unlikely to compromise the protein's stability and overall conformation, serving similarly to organelle localization motifs commonly employed in protein engineering. To minimize the inclusion of EC2, we aligned sequences of CD9, CD63 and CD81 (with validated TMs) and truncated CD63 after V111 keeping the conserved three hydrophilic amino acid residues to ensure its positioning out of the lipid membrane (Figure 7).

**Figure 7:**
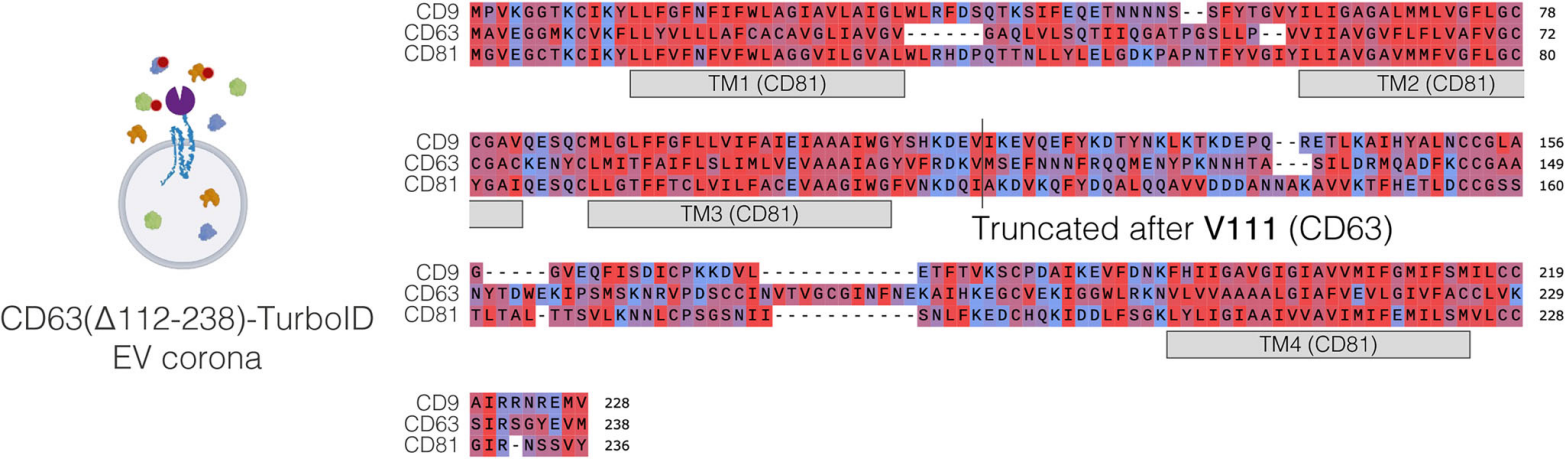
Introducing CD63(Δ112–238)‐TurboID. Protein alignment analysis of the three related tetraspanins CD9, CD63 and CD81 along with a validated crystallographic structure of transmembrane domains (TMs). The red and blue colours of the amino acid residues show their hydrophobicity where red represents hydrophobic amino acids typically clustered around the four TMs. The position of truncation is indicated after TM3.

We initially compared CD63‐TurboID (EC1) and CD63(Δ112–238)‐TurboID and found that CD63(Δ112–238)‐TurboID produces a markedly richer protein profile, yielding a higher number and greater abundance of detectable proteins (Figure 8A). Consequently, we chose CD63(Δ112–238)‐TurboID as the ‘EV corona’ construct replacing its prototype, and performed further experiments along with the other two control variants revealing distinct protein enrichment profiles at a comparable degree of biotinylation (Figure 8B). This suggests that TurboID fused to the truncated CD63 is more stably expressed with little impact on its enzyme activity.

**Figure 8:**
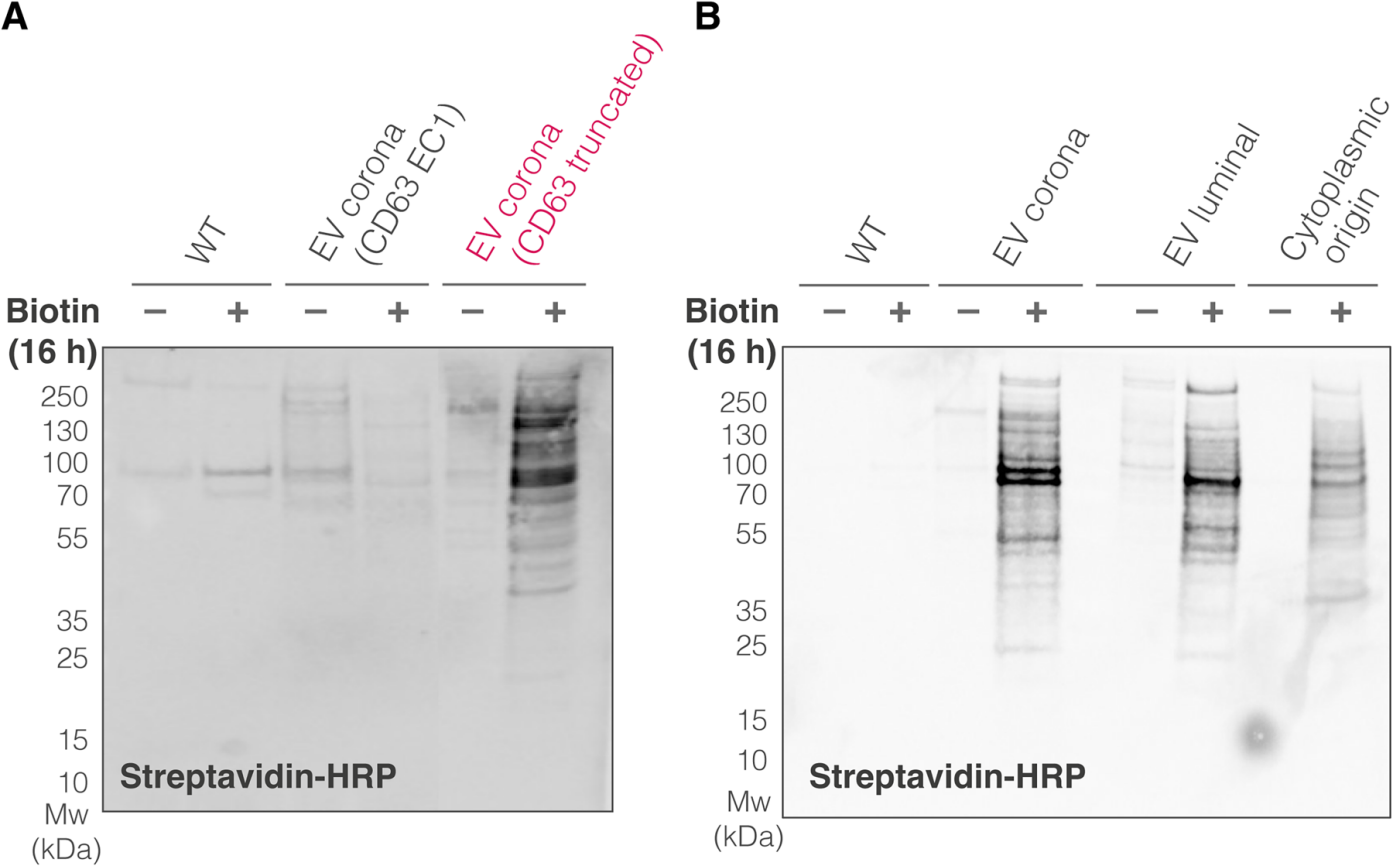
Replacing the prototype of the 'EV corona' construct with an enhanced fusion variant based on truncated CD63. (A) Western blot to compare CD63‐TurboID (EC1) and CD63(Δ112–238)‐TurboID. (B) Western blots to directly compare the three variants applying the same development time.

### 3.5 Characterization of Isolated EVs to Validate the Three Fusion Variants

To ensure that these differences were not attributable to variations in EV production, integrity or physicochemical properties, we performed characterization of the secreted EVs isolated by ultracentrifugation (Figure 9). To first confirm the expression of CD63‐TurboID fusion variants on EVs, we used anti‐CD63, anti‐TurboID and anti‐Flotillin‐1 antibodies in Western blots (Figure 9A, B). While CD63‐TurboID had a size corresponding to the fusion protein (∼75 kDa), we did not observe the same length for CD63‐TurboID (EC1), nor did we find any signals from CD63(Δ112–238)‐TurboID (Figure 9A). This absence of signal suggests that the epitope recognized by the anti‐CD63 antibody is located within the deleted EC2 region of the protein that is more variable among other tetraspanins (Figure 7). Furthermore, CD63‐TurboID (EC1) on isolated EVs, although its cellular pool had the correct size, revealed a band at a shorter size (∼40 kDa) than expected, likely due to improper folding and cleavage of the fusion protein often reported for genetically engineered EVs (Corso et al. 2019). Supporting the hypothesis that CD63(Δ112–238)‐TurboID is not detected due to the loss of the surface CD63 epitope, it was detected at the right size (∼50 kDa) when TurboID was targeted instead (Figure 9B). Notably, EC2 deletion resulted in a more discrete band on Western blot, as this large extracellular loop harbours multiple N‐glycosylation sites responsible for the characteristic smeared migration pattern typically observed for full‐length CD63. The other two variants were also found at the respective sizes, and in all conditions Flotillin‐1, used as an EV marker, was detected (Figure 9B). The identification of non‐fused TurboID on isolated EVs imply that overexpression of TurboID leads to passive loading into EVs possibly via plasma membrane‐derived EVs such as ectosomes and apoptotic bodies. Those isolated EVs also neither varied in the size distribution pattern nor in the particle concentration (∼10^10^ particles/mL), as evidenced by NTA, demonstrating a predominant population within the size range expected for small EVs (∼100–200 nm) (Figure 9C). Taken together, we concluded that expression of the CD63‐TurboID fusion variants does not affect the overall characteristics of secreted EVs and the observed differences in streptavidin Western blots can be attributed to where TurboID is localized.

**Figure 9:**
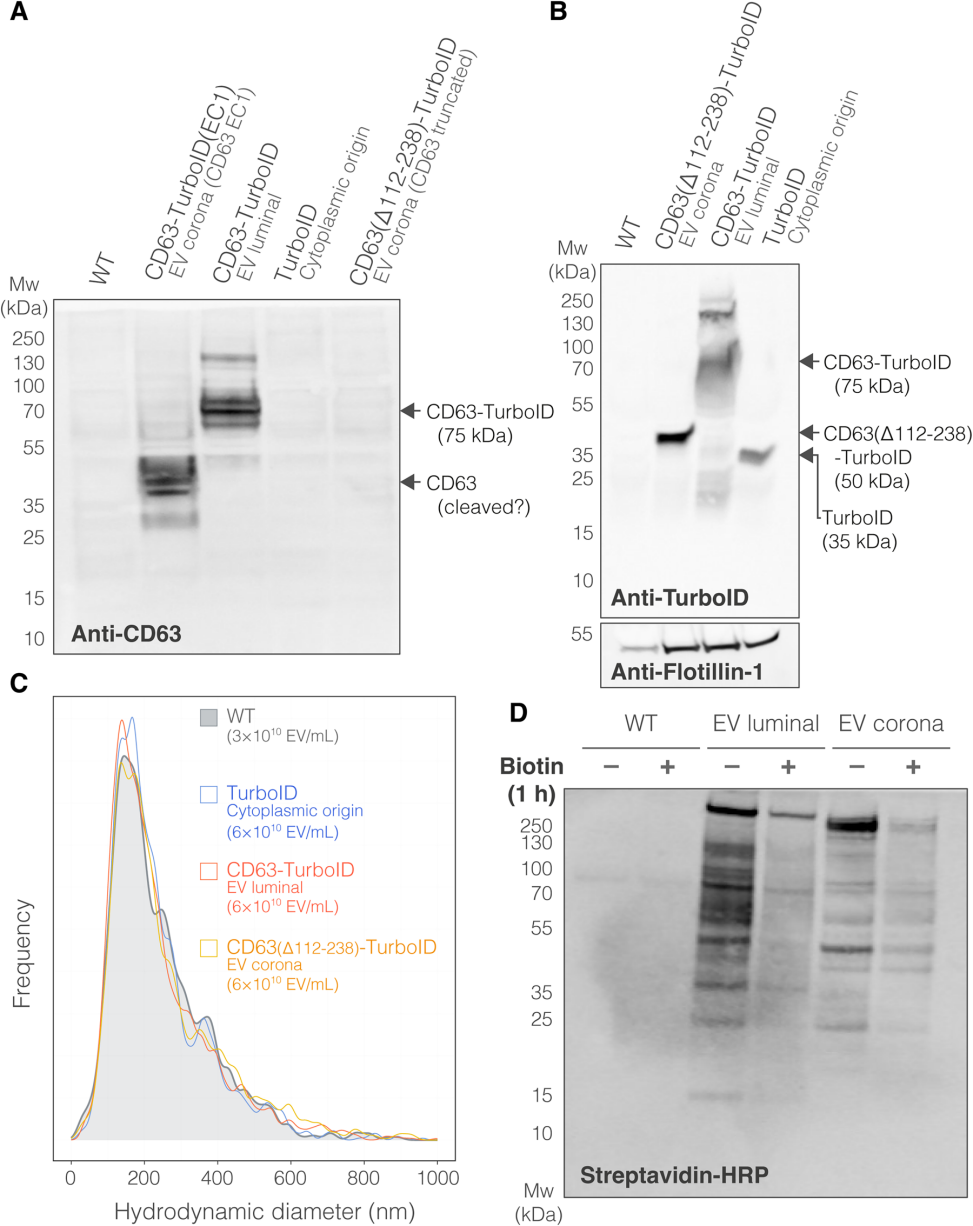
Characterization of isolated EVs following overexpression of the CD63‐TurboID fusion variants. (A) Western blot on anti‐CD63. (B) Western blots on anti‐TurboID (specific to overexpression) and anti‐Flotillin‐1 (as a general EV marker, showing only a single band without non‐specific signals). Note that a weaker Flotillin‐1 signal intensity was observed in WT consistent with the lower particle concentration estimated by NTA, likely resulting from a partial loss of the supernatant during the large‐scale EV isolation process. (C) Nanoparticle tracking analysis. (D) Ex situ proximity labelling on isolated EVs with biotin and ATP additions in the absence of cells. The constructs used in this experiment contain a V5 tag (14 AA) at the C‐terminus.

### 3.6 Ex Situ Proximity Labelling on Isolated EVs

To confirm that the TurboID presented at the EV surface functions properly as designed, we tested biotinylation *ex situ* only after isolation of EVs, that is, in the absence of the transfected cells. It should be noted, however, that a larger‐scale cell culture is necessary to achieve a sufficient number of EVs for the same level of biotinylation efficiency at the same serum concentration (10% FBS in this study). Isolation of EVs, especially using ultracentrifugation, may also impact the native EV architecture and the resulting activity of overexpressed TurboID fusion proteins. Here, we were able to provide a proof of concept that our approach can be applied to isolated EVs, albeit less efficiently (Figure 9D). In fact, higher levels of biotinylated proteins were observed in the biotin‐negative conditions, likely due to the presence of endogenous biotin. The excess biotin removal step applied only to the biotin‐treated samples leads to a partial loss of the total proteins (and/or EVs) as discussed above in the method optimization process. Interestingly, particularly strong signals were observed in EV luminal, presumably reflecting carryover of biotinylated proteins (labelled by endogenous biotin) that are retained in EVs during the transfection phase, whereas in EV corona free proteins labelled by endogenous biotin would have been all removed during the EV isolation process ‘resetting’ the cumulative biotinylated proteins. Nevertheless, the overall biotinylation patterns remained comparable between biotin‐treated and untreated samples, with differences being primarily quantitative rather than qualitative (Figure 9D). These results support the proper functionality of TurboID at the EV surface and indicate that each fusion variant generates a specific biotinylation profile reflective of its associated spatial proteome.

### 3.7 Five Functional Clusters of Biotinylated Proteins Reflect Spatially Resolved Proteomes

Mass spectrometry (MS) provides a powerful approach for the precise identification and quantitative characterization of the proteome, and when combined with proximity labelling it enables deeper functional insights into the bait protein's interaction footprint in a spatially resolved manner. To reliably analyse the different protein profiles by MS, we also optimized a protocol based on the use of S‐Trap columns (ProtiFi). This sample‐processing technology offers the major advantage of allowing the use of SDS while remaining fully compatible with downstream digestion and MS analysis steps. The S‐Trap matrix facilitates protein capture, cleanup, and digestion under highly denaturing conditions, thereby improving peptide preparation efficiency and analysis reproducibility. This protocol therefore provides a robust approach for the comparative study of proteomic signatures. Here we performed label‐free quantification based on spectral intensity of the precursor ions that correlate with unique peptide abundance and thus respective proteins. Clustering analysis revealed distinct protein profiles across the CD63‐TurboID fusion variants underscoring the results obtained by Western blotting (Figure 10A). The EV corona condition was clearly enriched in proteins exclusively found in this fusion variant that characterize Cluster 1, and to a lesser extent, Cluster 2, but also highly enriched in other proteins that are shared by the two control fusion variants such as Clusters 3 and 4, whereas it is significantly depleted of proteins in Cluster 5 (Figure 10B, C). The Cytoplasmic origin condition showed enrichment in Clusters 3 and 4 and depletion in Clusters 1 and 2, while the EV luminal condition had a characteristic suppression of Cluster 4 proteins (Figure 10B, C). Based on the non‐scaled abundance data, EV corona does in general have significantly higher abundance of those proteins in Clusters 1–4, but clearly lower abundance of the Cluster 5 proteins among others (Figure 10C). The cluster analysis was performed on the dataset with a cut‐off threshold to exclude low abundance proteins as it increases the fraction of the extreme cases (high z‐scores due to zero detection of the protein in other samples) if no threshold is applied (Figure S1). Looking closer to the individual proteins identified and where they originate, both *Homo sapiens* and *Bos Taurus* are found representing those from the cells themselves and those from the bovine serum supplemented to the cell culture medium (Table S2). To characterize the functional organization of the identified protein sets, a STRING protein–protein interaction network analysis was performed for each cluster, separately for human and bovine origins. Gene Ontology analysis then gave insights into biological processes, molecular functions, and cellular components, refining the functional interpretation of the EV corona footprint (Figures 11, 12, 13).

**Figure 10:**
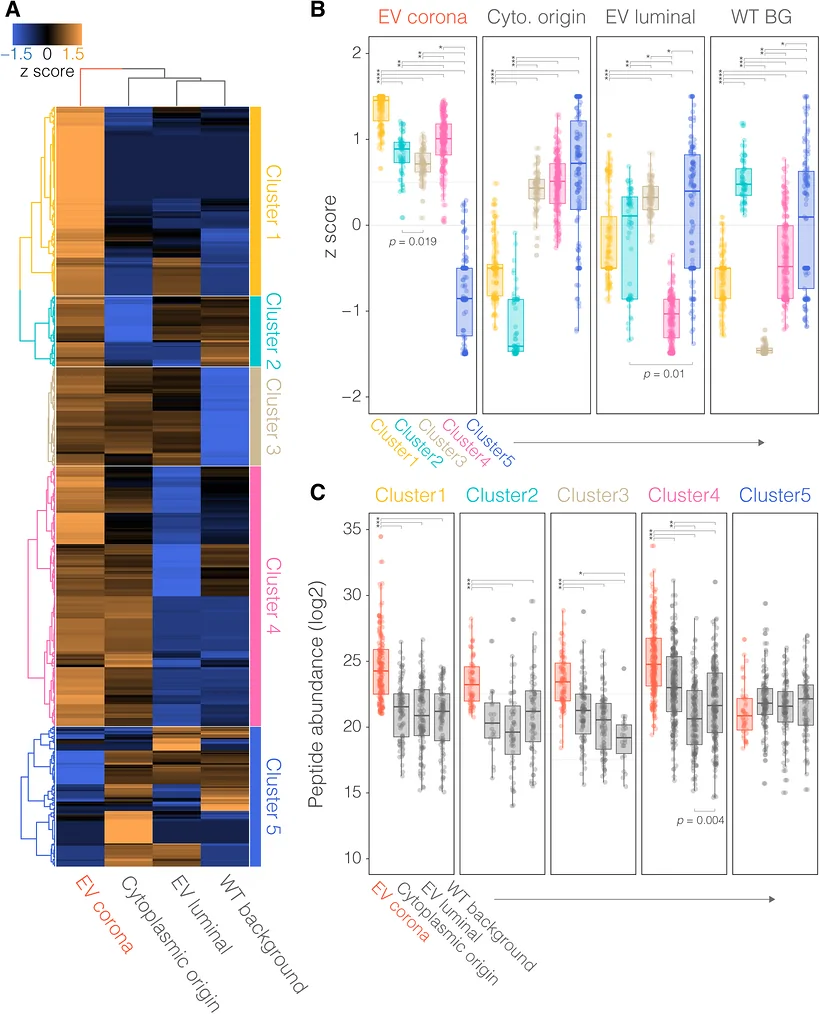
Proteomic clustering reveals distinct EV‐protein corona signature. (A) The relative abundance (z‐score) of each detected protein across the four experimental conditions (CD63(Δ112–238)–TurboID (EV corona), TurboID (Cytoplasmic origin), CD63–TurboID (EV luminal), and WT background is shown as a heatmap together with a two‐way unsupervised hierarchical clustering analysis. (B) Z‐score distribution of each protein grouped into five clusters (Clusters 1–5) across the different fusion variants. (C) Peptide abundance (log2‐transformed) of respective proteins belonging to each cluster across the fusion variants. Asterisks denote a significant difference at adjusted *p*‐value < 0.001, while an exact *p*‐value is indicated when 0.001 < adjusted *p*‐value < 0.05 (Welch's ANOVA with Games‐Howell post hoc tests).

**Figure 11:**
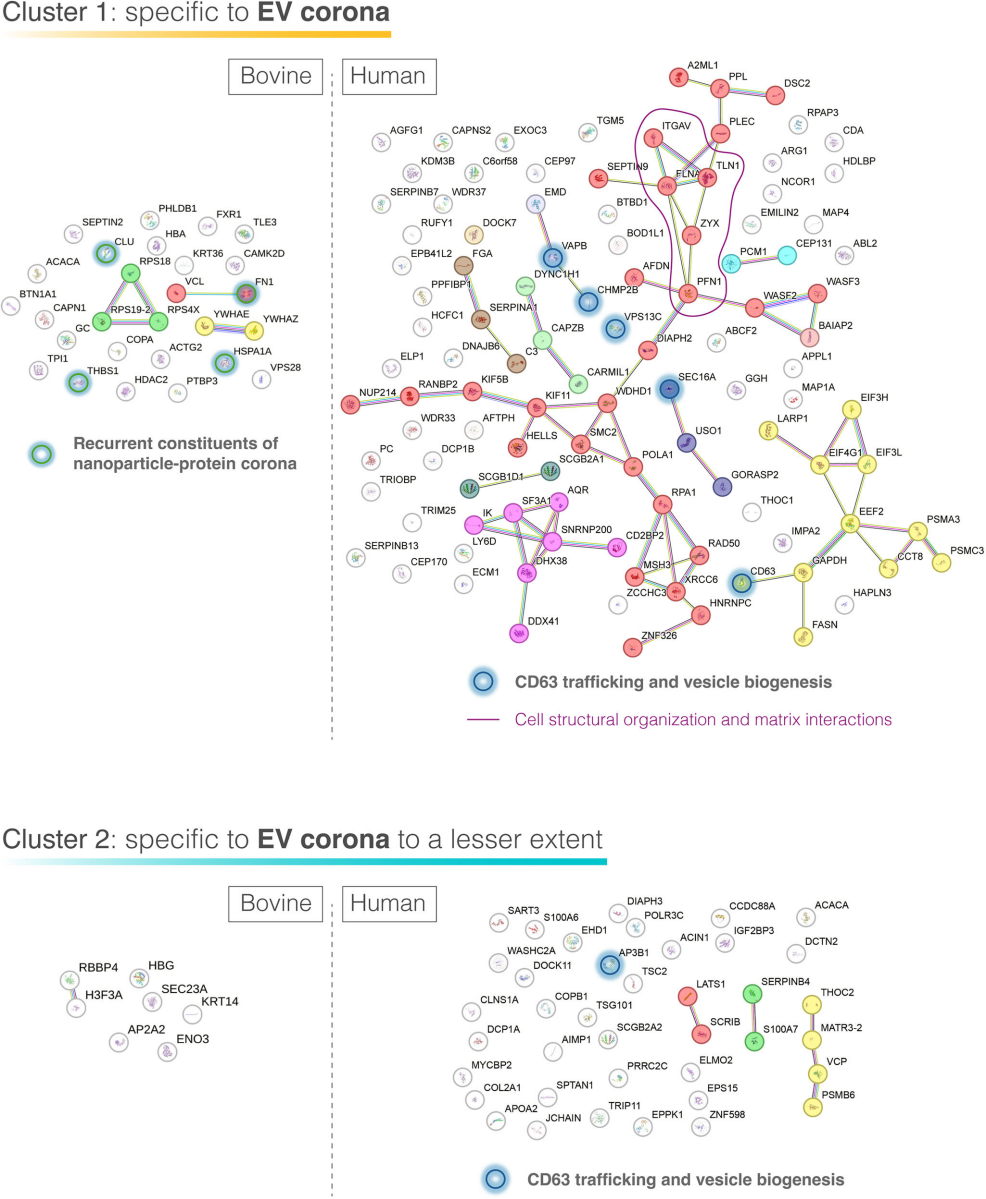
STRING network of functional interactions among proteins enriched in clusters specific to EV corona. Nodes represent proteins identified in our proteomic analysis, and edges indicate high‐confidence functional associations derived from experimental evidence, curated databases, and co‐occurrence.

**Figure 12:**
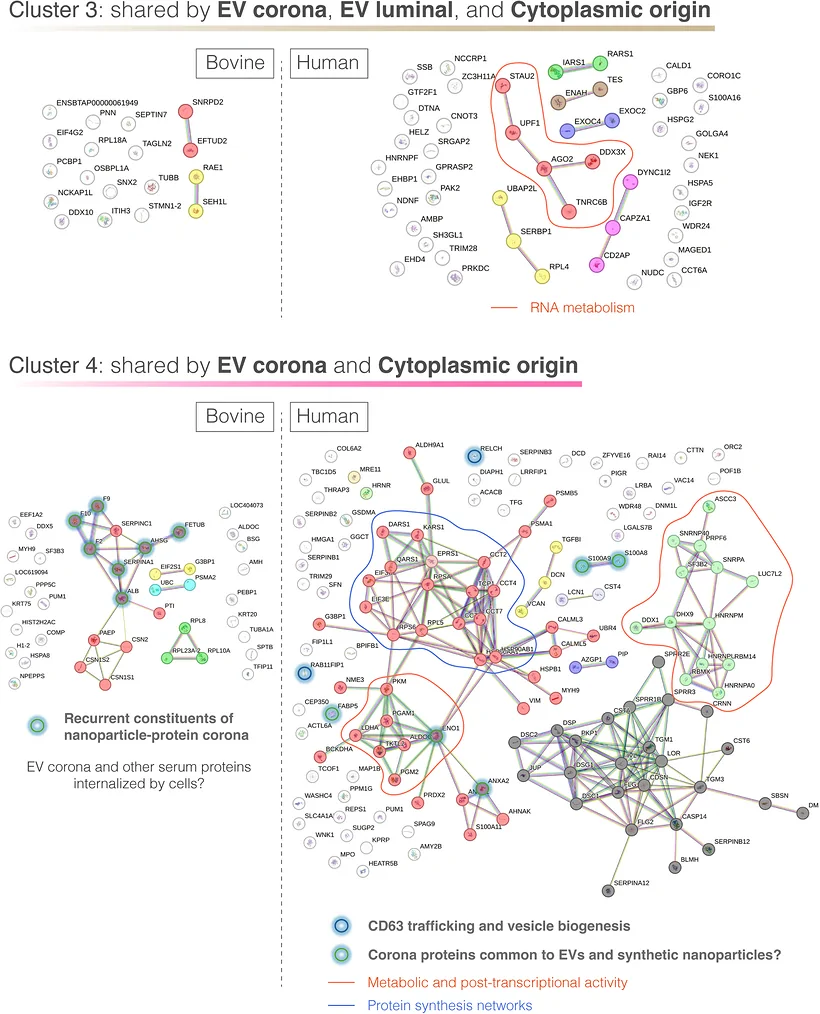
STRING network of functional interactions among proteins enriched in clusters common to EV corona and Cytoplasmic origin.

**Figure 13:**
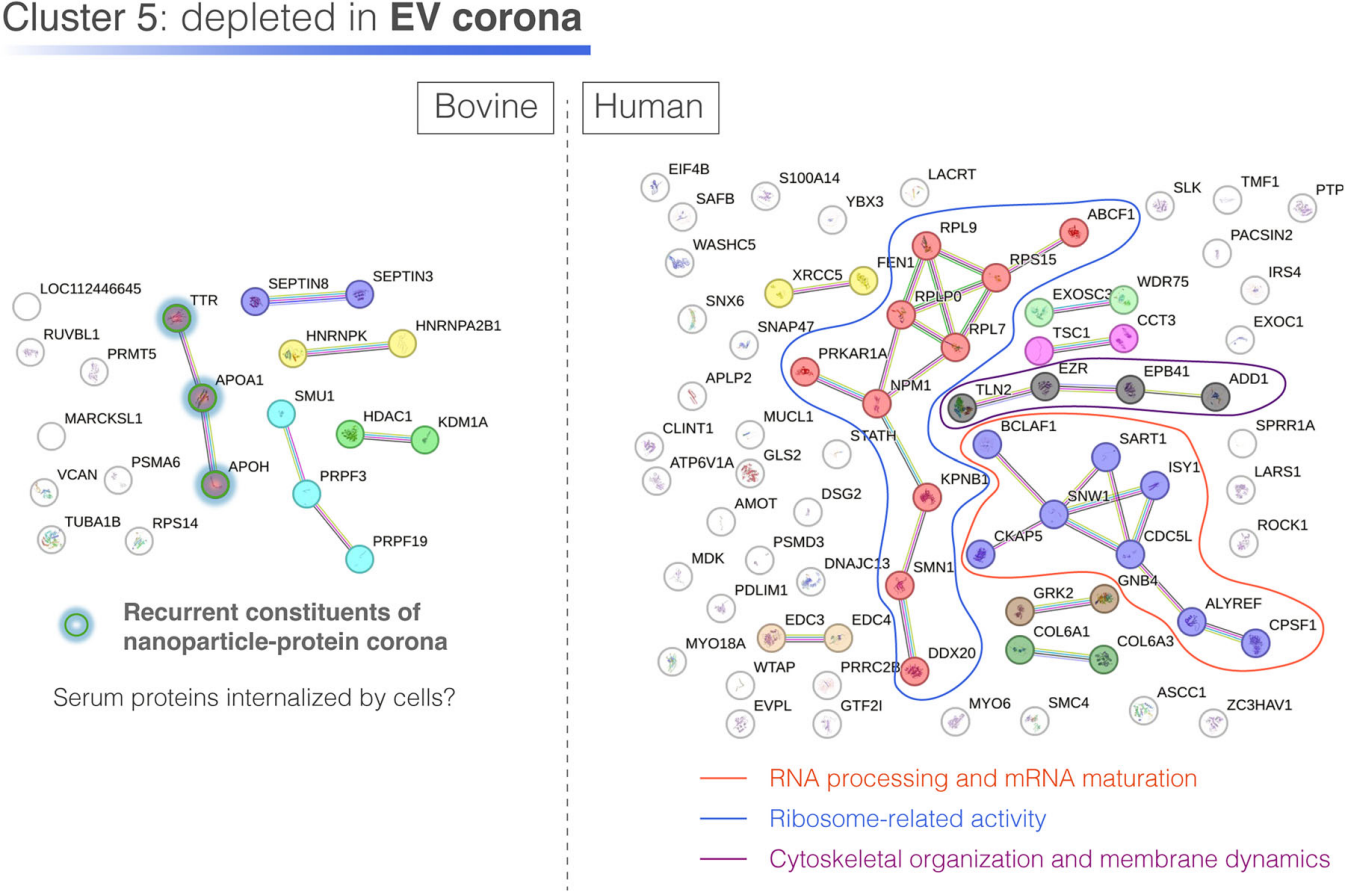
STRING network of functional interactions among proteins enriched in Cluster 5 depleted in EV corona.

**Clusters 1 and 2** represent proteins that may characterize the EV corona, both endogenous and acquired in the extracellular space. Among these, canonical EV‐associated proteins were identified including the exosomal marker CD63 (supporting co‐localization with the truncated CD63) and charged multivesicular body protein 2B (CHMP2B), a component of the endosomal sorting complex required for transport (ESCRT) implicated in EV biogenesis. Additional trafficking‐related proteins involved in CD63 dynamics, such as protein transport protein SEC16A, VAMP‐associated protein B/C (VAPB), vacuolar protein sorting‐associated protein 13C (VPS13C) and AP‐3 complex subunit beta‐1 (AP3B1) were also enriched in these clusters (Figure 11). Of particular interest is a functionally coherent interactome structured into several major biological pathways. One prominent module comprised cytoskeletal and adhesion‐associated proteins, including FLNA, TLN1, ITGAV and PFN1, suggesting roles in mechanical structuring and cell–matrix interactions (Figure 11). Bovine proteins that belong to these clusters were, on the other hand, less interconnected and likely represent biophysical interactions at the EV surface. For example, clusterin (CLU), fibronectin (FN1), Hsp70 family chaperone (HSPA1A), thrombospondin‐1 (THBS1) have previously been reported as recurrent constituents of protein corona formed when synthetic nanoparticles are exposed to biological fluids (Tassoni et al. 2025, Monopoli et al. 2012). Proteins in **Clusters 3 and 4** were common to EV corona and Cytoplasmic origin, possibly reflecting the intracellular proteome, with the luminal side of limiting membranes (EV luminal) is enriched with Cluster 3 proteins (Figure 12). The network identified in this cluster represents a central module involved in post‐transcriptional regulation and RNA metabolism, centred on the AGO2–TNRC6B–UPF1–DDX3X axis. As cells were carefully removed through differential centrifugation prior to IP and free TurboID was detected in isolated EVs (Figure 9D), in practice proteins detected in Cytoplasmic origin represent biotinylated proteins secreted into the extracellular space directly, or indirectly as the EV cargo. Unlike Clusters 1 and 2, Cluster 4 exemplifies highly expressed cellular proteins such as ribosomal proteins, translation factors, RNA binding proteins, and glycolytic enzymes linked to the translational and metabolic state of the producer cells (Figure 12). Among proteins involved in vesicle trafficking, we also identified Rab11 proteins (RAB11FIP1, RELCH) that have a role in endosome recycling (Figure 12). The implications are that these proteins may result from very weak and transient interactions with CD63 during EV biogenesis (or stochastically when expressed as free TurboID) and passive loading of the biotinylated proteins into EVs, especially of those originating from the plasma membrane budding. On the other hand, many of the bovine proteins identified in Cluster 4 belong to typical serum components abundant in FBS such as albumin (ALB), fetuins (AHSG, FETUB), serine protease inhibitors of the serpin family (SERPINA1, SERPINC1) as well as coagulation factors (F2, F9, and F10). These proteins are likely to dynamically interact with EV surfaces in a similar manner to cell membranes, also as a classical corona protein at synthetic nanoparticles. The high abundance may also play a role in Cytoplasmic origin where those proteins are first taken up and encounter free TurboID along intracellular trafficking of extracellular serum proteins rather than simple extracellular contamination (Frei 2011, Jaldín‐Fincati et al. 2019). Note that **Cluster 5** encompasses proteins that were depleted in EV corona, revealing general processes of cell physiology (e.g. ribosomal proteins, nucleolar factors, RNA processing and spliceomal proteins, cytoskeletal components, and extracellular matrix proteins) (Figure 13) at an abundance level comparable to that of the WT background control (Figure 10C). Interestingly, apolipoproteins (APOA, APOH) and transthyretin (TTR) that are typically found in the protein corona of synthetic nanoparticles were found in this cluster negative for EV corona.

## 4 Discussion

### 4.1 Development of a Proximity Biotinylation Protocol Adapted to the Extracellular Space

This study aimed to establish a robust protocol for mapping the protein corona of EVs by proximity biotinylation. Our results indicate that direct application of original TurboID protocol, optimized for the intracellular environment, to the extracellular milieu is ineffective. We specifically demonstrate that the presence of excess free biotin in the supernatant constitutes a major obstacle: unlike intracellular approaches, where biotin can be removed by washing, it persists in the cell culture medium and rapidly saturates streptavidin beads, thereby preventing the capture of biotinylated proteins. Introducing a column‐based free biotin removal step effectively solved this issue, confirming that controlling the pool of unreacted biotin is an essential prerequisite for any extracellular application of TurboID. Another limitation identified concerns TurboID's dependence on ATP. Indeed, biotin ligase approaches built on BioID use ATP to generate a reactive intermediate that labels neighbouring proteins (Branon et al. 2018, Li et al. 2023). This contrasts them with other similar methods such as peroxidase‐based APEX2 which do not require ATP—they catalyse the oxidation of biotin‐phenol substrates in the presence of hydrogen peroxide to generate short‐lived radicals that enable rapid labelling, even in low‐energy environments (Ahamed et al. 2022), but less suitable for applications beyond cells (Guo et al. 2023). TurboID is compatible with living systems, but their efficiency may vary in compartments or conditions where ATP levels are reduced or heterogeneous (Chen et al. 2017). We show that supplementing the cell culture medium with ATP at a concentration compatible with cell viability (50 µM) noticeably improves labelling efficiency. These observations emphasize that the biochemical parameters of the extracellular space must be carefully considered and adjusted to ensure optimal enzyme activity.

### 4.2 Structural Constraints Related to CD63 Engineering and Implications for Biotinylation

The prototype construct using CD63 as a bait for proximity biotinylation was inspired by previous studies showing that the insertion of a reporter protein into the EC1 loop is compatible with CD63 trafficking and incorporation into EVs (Verweij et al. 2018). However, our results indicate that this strategy cannot be directly adapted when the fused protein is as large as TurboID (∼35 kDa) with its N‐ and C‐termini are widely separated, unlike the compact proteins such as pHluorin. Indeed, the low level of labelling obtained with CD63‐TurboID (EC1), despite an optimized protocol, suggests that the enzyme introduces steric constraints that may disrupt its presentation on the EV surface or affect CD63 folding. To improve the biotinylation efficiency, we truncated the distal portion of the EC2 loop, which is more flexible and better tolerates modifications without compromising protein structure or function, making it a more suitable engineering target (Curley et al. 2020, Nørgård et al. 2022). Compared to CD63‐TurboID (EC1), our results demonstrate that the CD63(Δ112–238)‐TurboID construct exhibits remarkably higher activity, producing both substantial and reproducible proximity biotinylation. Given that the fused protein correctly localizes to the EV membrane, this underscores the use of truncated tetraspanins as a particularly effective platform for displaying functional proteins on EVs. It should be noted, however, that regardless of the TurboID insertion site on CD63, not all proteins at the EV surface is expected to be biotinylated; nevertheless, the high copy number of CD63 found on EVs (Corso et al. 2019) ensures that a meaningful fraction of proteins interacting at the EV surface can be labelled. Indeed, CD63 is preferentially localized to EV membranes, often exhibiting higher abundance than at the plasma membrane, as shown by quantitative proteomic studies (Kowal et al. 2016, Jeppesen et al. 2019). Importantly, our method can be extended to other EV‐associated proteins as a bait to map additional subsets of the EV corona proteome, as demonstrated in a recent study although in a different context, enabling systematic exploration of distinct EV microenvironments (Li et al. 2023).

### 4.3 Decoding the Spatial Proteomic Signature of EV Corona

The main goal of this study was to identify all proteins that were in proximity to or interacted with EVs as corona footprint. Performing EV isolation, for example by ultracentrifugation, could disrupt the endogenous protein corona and alter the protein‐protein interactions when introduced to a new protein environment. To preserve both the endogenous EV corona and any additional proteins acquired in the extracellular space without perturbing their native context, here we argue for the advantage of in situ proximity labelling in the presence of EV donor cells to map spatially resolved signature of proteins interacting with CD63. Supporting our rationale to focus on CD63 as the bait protein in the endogenous approach, as described and demonstrated in several studies, CD63 is strongly enriched in small EVs compared with larger vesicular populations such as ectosomes. On this premise, we isolated EVs from transfected cells and characterized the fusion variants: CD63(Δ112–238)‐TurboID (‘EV corona’), CD63‐TurboID (‘EV luminal’) and free TurboID (‘Cytoplasmic origin’) that did not vary in the size distribution and EV concentration but did in how TurboID was presented on EVs. Notably, the free TurboID ‘Cytoplasmic origin’ control revealed its presence in isolated EVs likely due to passive loading of (overexpressed) cytosolic proteins into EVs. After confirming TurboID's activity *ex situ*, we performed LC‐MS/MS on captured biotinylated proteins isolated from the cell culture supernatant. Instead of comparing the number of proteins identified in each condition, here we opted for label‐free quantification to exclude low abundance proteins and focus on differential enrichment of the spatially resolved protein profiles. This is also because TurboID biotinylates a broad array of nearby proteins not necessarily with a biological context (that may be biased towards highly abundant proteins), and biotinylation itself is not a proof of direct protein‐protein interactions. Overall, the cluster‐based STRING analysis revealed spatially‐defined organization of both human and bovine protein networks associated with EVs and other cellular components. The EV corona footprint signified the engagement of intracellular proteins recruited during EV biogenesis, in particular vesicle trafficking (CD63, SEC16A, CHMP2B, VAPB, VPS13C, RAB11FIP1, RELCH and AP3B1) (Colombo et al. 2014, Urwin et al. 2010, Sprangers and Rabouille 2015, Rous et al. 2002, Menaceur et al. 2021, Pols and Klumperman 2009), highlighting the interaction history of the bait protein—CD63 — from its translation in ER to maturation of the EV in late endosomes (Figure 14). Human interactomes displayed coherent modular architectures enriched in RNA metabolism, translation and cytoskeletal organization, potentially reflecting biologically meaningful cargo selection mechanisms that contribute to the endogenous formation of a protein corona around the EV. Of particular interest are cytoskeletal and adhesion proteins (FLNA, TLN1 and ITGAV) that reinforce the concept of tight functional coupling between cytoskeletal architecture, endosomal trafficking, and EV biogenesis. Previous studies have shown that ESCRT machinery and actin networks cooperate during multivesicular body formation and small EVs secretion (Colombo et al. 2014). Our data raise the intriguing possibility that the protein enrichment observed with the EV corona construct reveals not only the intrinsic EV proteome but also features reminiscent of a protein corona like that described for synthetic solid nanoparticles (Table 1). Indeed, several human proteins enriched under this condition, including S100A8/S100A9 (often as the heterodimer calprotectin), annexin A2 (ANXA2), and fatty‐acid binding protein 5 (FABP5), correspond to cargos classically identified in circulating EVs and reported in multiple protein corona studies across diverse biological contexts. Among bovine proteins, clusterin is highly relevant in understanding the interaction of the corona with cells. It is a secreted chaperone, well‐described as a dysopsonin, known to associate with synthetic nanoparticles reducing cellular uptake by limiting interactions with cell surface receptors, thereby prolonging circulation half‐life as ‘stealth’ (Schöttler et al. 2016). Concurrently, other enriched proteins (both in humans and bovine), such as albumin (ALB) and enolase 1 (ENO1/3), are well‐established components of a protein corona formed on synthetic nanoparticles upon exposure to biological fluids (Jeppesen et al. 2019, Ahmadi et al. 2025, Zhang et al. 2024, Tsamchoe et al. 2023, Salmond et al. 2025, Singh et al. 2023). This dual signature, combining a core EV biogenesis‐associated proteome and classical nanoparticle corona proteins, suggests that EVs (like synthetic nanoparticles) may acquire a dynamic biological identity underlining protein‐protein interactions upon exposure to extracellular milieus such as plasma.

**Figure 14:**
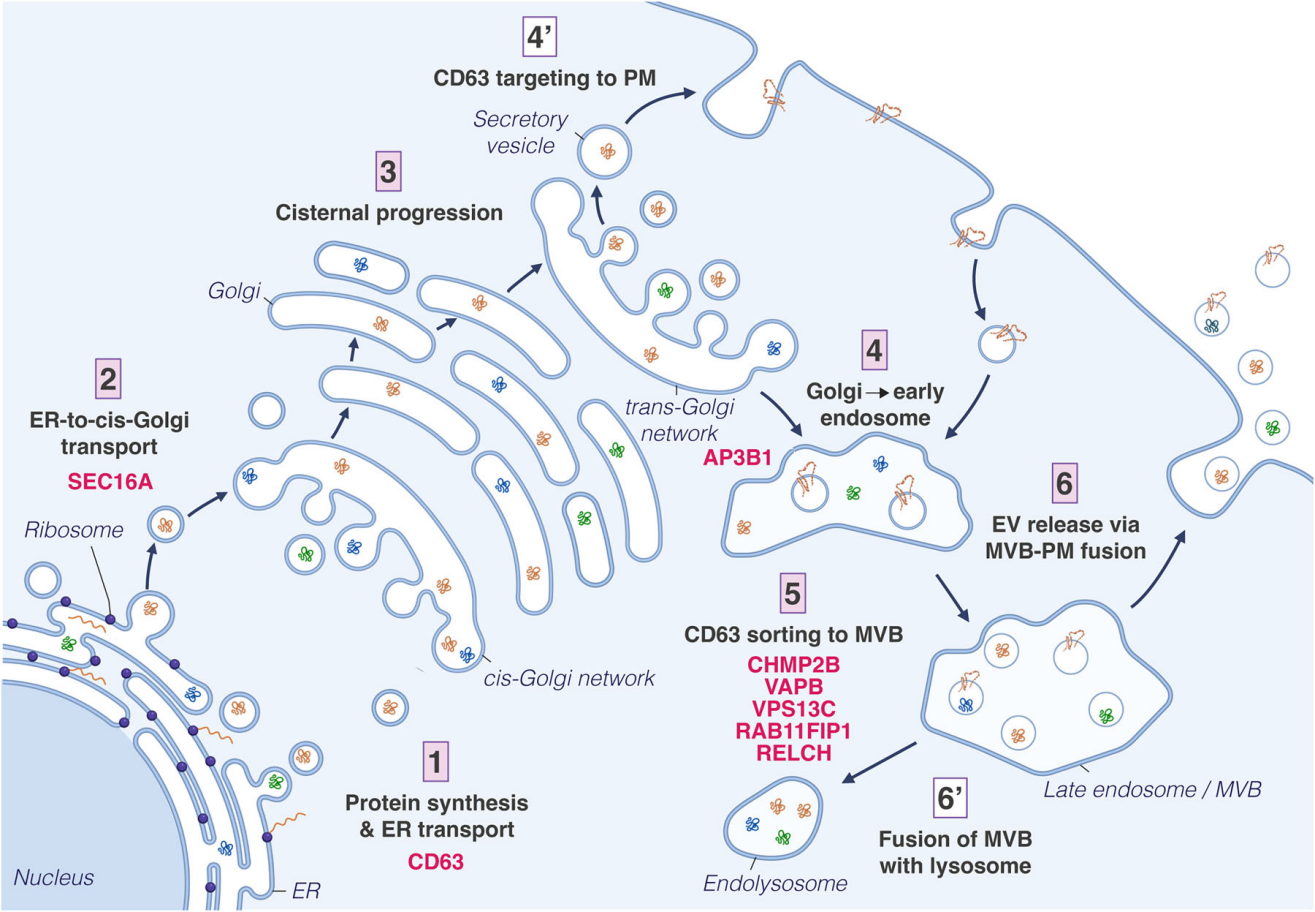
EV corona proteins identified as involved in the intracellular trafficking of CD63. Diagram illustrating the different stages of CD63 trafficking from its synthesis in the endoplasmic reticulum to its targeting to endosomal compartments and its release via EVs. 1. CD63 is synthesized in the ER, where SEC16A participates in the organization of ER exit sites and transport to the Golgi apparatus. 2. ER–cis‐Golgi transport followed by cisternal progression through the Golgi apparatus 3. Cisternal progression of the proteins. 4. At the trans‐Golgi network (TGN), the AP3B1 adaptor complex contributes to the sorting of CD63 to the early endosomal pathway. CD63 can also be addressed directly to the plasma membrane via secretory vesicles (4’). 5. The sorting of CD63 to multivesicular bodies (MVBs) involves several regulators of vesicular traffic, including RAB11FIP1, RELCH, VPS13C, VAPB and CHMP2B (ESCRT machinery), which participate in the formation of intraluminal vesicles. 6. MVBs can either fuse with the plasma membrane to release EVs or fuse with lysosomes for degradation of their contents (6’). Proteins shown in red and bold were enriched in the EV corona condition. ER: endoplasmic reticulum; MVB: multivesicular body; PM: plasma membrane; Figure created with BioRender.com.

**Table 1:** Proteins identified in the EV corona condition that are common to EV proteomes and nanoparticle‐protein coronas. The listed proteins have been previously identified in proteomic analyses of EVs and/or nanoparticle‐protein coronas, supporting that the EV corona proteins observed here reflects a molecular microenvironment typical of EV biogenesis pathways and protein–Surface interactions.

Protein	EV link	PC link	Reference
Albumin (ALB)	Present in EV fraction	Major protein in nanoparticle coronas	(Guglielmelli et al. 2023) (Ahmadi et al. 2025)
Annexin A2 (ANXA2)	Very common in EVs	Described in nanoparticle–membrane interfaces	(Jeppesen et al. 2019) (Oberländer et al. 2025)
S100A8	EV cargo, enriched in pathological EVs	Involved in surface–lipid interactions	(Hadjidemetriou et al. 2019) (Xin et al. 2023) (Zhang et al. 2024)
S100A9	Strong association with circulating EVs	Combined with surface adsorption/corona	(Tsamchoe et al. 2023) (Xin et al. 2023)
Enolase 1/3 (ENO1/3)	Very often identified in EV	Observed in corona‐type environments	(Mosquera et al. 2020) (Salmond et al. 2025)
Fatty‐acid Binding protein 5 (FABP5)	Described in EVs (urine, cancer)	Corona‐compatible lipid interaction	(Fujita et al. 2017) (Singh et al. 2023)

### 4.4 Revisiting the ‘Hard’ Versus ‘Soft’ Concept of the Protein Corona

Although the distinction between ‘hard’ and ‘soft’ corona components remains debated even after more than a decade of research in the bionanoscience field, one might consider a less rigid model in which the same protein can exhibit differential binding states, ranging from transient (‘soft’) to progressively stabilized (‘hardening’) interactions, as a function of time and environmental conditions (Mohammad‐Beigi et al. 2020a). Plasma‐abundant proteins such as albumin or immunoglobulins may preferentially engage in softer, more dynamic interactions at the EV surface in biofluids, whereas kinetically trapped and more stably associated proteins (e.g., annexins or S100 family members) could represent a harder corona or even constitutive EV membrane/peri‐membrane components (Mohammad‐Beigi et al. 2020a, Xin et al. 2023). Importantly, the proximity labelling approach should, in principle, label these proteins equivalently through biotin tagging, independently of interaction strength, in contrast to physical isolation strategies that are likely biased toward capturing predominantly hard interactions. In this respect, our approach offers a conceptual and methodological advantage by bridging intrinsic EV biogenesis with the extrinsic acquisition of a protein corona. It provides a refined view of EV identity, defined not only by their intracellular origin and cargo sorting mechanisms, but also by the dynamic, fluid‐dependent protein milieu encountered after secretion. This hybrid identity may critically influence EV stability, biodistribution, cellular uptake, and functional outcomes, aspects that warrant careful consideration in EV research, particularly when extrapolating in vitro preclinical findings to in vivo and clinical settings.

## 5 Limitations and Perspectives

To further advance this study, several limitations should be considered, some of which are currently being addressed to confirm the robustness and specificity of our approach. TurboID's enzymatic activity remains dependent on exogenous ATP, which may limit certain in vivo applications; however, studies in *Drosophila* have demonstrated its effectiveness in capturing transient interactions in living organisms (Branon et al. 2018, Vianney and Lee 2025), highlighting its utility for identifying proximal proteins in complex biological contexts. A limitation to consider when using TurboID is the potential detection of proteins that are not directly related to the biological compartment of interest. For instance, nuclear or cytosolic proteins accounted for >75% of biotinylated proteins labelled by TurboID localized to mitochondria, likely a result of the fusion protein being active even before it reaches the target organelle (Yan et al. 2023). It is therefore encouraged to compare TurboID fusion variants to identify a characteristic signature not shared by the common background proteome. Observed differences between our CD63‐TurboID constructs underscore the importance of molecular design, leaving room to explore alternative tetraspanins, insertion sites, and linker length as well as flexibility. Combining TurboID with more refined spatial targeting strategies or orthogonal EV isolation approaches may help to further disentangle intracellular versus extracellular contributions and improve specificity toward EV surface‐associated proteins. Biotin incubation times will need optimization to minimize potential cytotoxicity in vivo (Branon et al. 2018), although our results show that TurboID can be effective after just 10 min. Given TurboID's compatibility with in vivo applications (Xiong et al. 2021), we plan to extend this approach to establishing a zebrafish model, in which the endogenous corona of proteins formed on EVs will be mapped entirely in situ to explore how an EV corona takes shape in vivo in a living organism. Highlighting the in situ directed approach of the spatial proteomics to map the EV corona footprint, we term this method ‘EV‐SPEC (Spatial Proteomics of Endogenous Corona)’ and envision expansion of the toolbox as a complementary approach to the physical EV corona isolation.

## Author Contributions

**Camille Menaceur Vandenbroucke**: investigation, writing – original draft, writing – review and editing, visualization, methodology, formal analysis, data curation, resources. **Carsten Scavenius**: investigation, methodology, writing – review and editing, formal analysis, supervision, resources, data curation. **Yuya Hayashi**: conceptualization, investigation, funding acquisition, writing – original draft, writing – review and editing, visualization, methodology, formal analysis, project administration, resources, supervision, data curation.

## Conflicts of Interest

The authors declare no conflicts of interest.

## Supporting information




**Supporting Information**: jex270151‐sup‐0001‐figureS1.tif

**Supporting Information**: jex270151‐sup‐0001‐tableS1.xlsx

**Supporting Information**: jex270151‐sup‐0001‐tableS2.xlsx

## Data Availability

The data that supports the findings of this study are available in the supplementary material of this article
